# Synergism and Antagonism of Two Distinct, but Confused, Nrf1 Factors in Integral Regulation of the Nuclear-to-Mitochondrial Respiratory and Antioxidant Transcription Networks

**DOI:** 10.1155/2020/5097109

**Published:** 2020-11-16

**Authors:** Shuwei Zhang, Yangxu Deng, Yuancai Xiang, Shaofan Hu, Lu Qiu, Yiguo Zhang

**Affiliations:** ^1^The Laboratory of Cell Biochemistry and Topogenetic Regulation, College of Bioengineering and Faculty of Medical Sciences, Chongqing University, No. 174 Shazheng Street, Shapingba District, Chongqing 400044, China; ^2^Department of Biochemistry and Molecular Biology, College of Basic Medical Sciences, Southwest Medical University, Sichuan 646000, China; ^3^School of Life Sciences, Zhengzhou University, No. 100 Kexue Avenue, Zhengzhou, 450001 Henan, China

## Abstract

There is hitherto no literature available for explaining two distinct, but confused, Nrf1 transcription factors, because they shared the same abbreviations from nuclear factor erythroid 2-related factor 1 (also called Nfe2l1) and nuclear respiratory factor (originally designated *α*-Pal). Thus, we have here identified that Nfe2l1^Nrf1^ and *α*-Pal^NRF1^ exert synergistic and antagonistic roles in integrative regulation of the nuclear-to-mitochondrial respiratory and antioxidant transcription profiles. In mouse embryonic fibroblasts (MEFs), knockout of *Nfe2l1^−/−^* leads to substantial decreases in expression levels of *α*-Pal^NRF1^ and Nfe2l2, together with TFAM (mitochondrial transcription factor A) and other target genes. Similar inhibitory results were determined in *Nfe2l2^−/−^* MEFs but with an exception that both *GSTa1* and *Aldh1a1* were distinguishably upregulated in *Nfe2l1^−/−^* MEFs. Such synergistic contributions of Nfe2l1 and Nfe2l2 to the positive regulation of *α*-Pal^NRF1^ and TFAM were validated in *Keap1^−/−^* MEFs. However, human *α*-Pal^NRF1^ expression was unaltered by *hNfe2l1α^−/−^*, *hNfe2l2^-/-ΔTA^*, or even *hNfe2l1α^−/−^+siNrf2*, albeit TFAM was activated by Nfe2l1 but inhibited by Nfe2l2; such an antagonism occurred in HepG2 cells. Conversely, almost all of mouse Nfe2l1, Nfe2l2, and cotarget genes were downexpressed in *α-Pal^NRF1+/-^* MEFs. On the contrary, upregulation of human Nfe2l1, Nfe2l2, and relevant reporter genes took place after silencing of *α*-Pal^NRF1^, but their downregulation occurred upon ectopic expression of *α*-Pal^NRF1^. Furtherly, Pitx2 (pituitary homeobox 2) was also identified as a direct upstream regulator of Nfe2l1 and TFAM, besides *α*-Pal^NRF1^. Overall, these across-talks amongst Nfe2l1, Nfe2l2, and *α*-Pal^NRF1^, along with Pitx2, are integrated from the endoplasmic reticulum towards the nuclear-to-mitochondrial communication for targeting TFAM, in order to finely tune the robust balance of distinct cellular oxidative respiratory and antioxidant gene transcription networks, albeit they differ between the mouse and the human. In addition, it is of crucial importance to note that, in view of such mutual interregulation of these transcription factors, much cautions should be severely taken for us to interpret those relevant experimental results obtained from knockout of Nfe2l1, Nfe2l2, *α*-Pal or Pitx2, or their gain-of-functional mutants.

## 1. Introduction

In all life forms, distinct types of cells are the most basic unit of their biological structures and functions, due to a fact that they are bounded by the membrane lipid bilayers and hence protected from extracellular oxidizing environments to which they have adapted. Of note, eukaryotic cells are evolutionarily compartmentalized by endomembrane systems, as a unique trait that distinguishes from prokaryotic cells [1], insofar as to give rise to distinct functionally specialized organelles, such as the mitochondria, endoplasmic reticulum (ER), and continuous membrane-surrounded nucleus in eukaryotic cells [2]. Such great benefits can allow for cellular respiration, redox metabolism, and biochemical reactions to take place properly in various temporospatial order during diverse physio-pathological life processes. This is owing to the fact that such versatile organelles participate in a large number of distinct cellular functions. Amongst them, the mitochondrion is known as a central site of cellular oxidative respiration, redox metabolism, and biosynthesis, in order to meet a host of energy and growth demands [3, 4].

Of particular concern is the mitochondrial origin from aerobic *α*-proteobacterium, which was firstly surmised to be engulfed by a primordial anaerobic eukaryote (i.e., the endosymbiont hypothesis [5]) or originally merged with its archaeal host to give rise to the first eukaryotic cell (i.e., the standpoint of comparative genomics [6, 7]). Thereby, this organelle has its own genetic system with the bacteria-like features, including a compact circular mtDNA genome (from maternal inheritance), along with a simple transcription system that yields multigenic RNA transcripts, and a translational apparatus with similar antibiotic sensitivities to prokaryotic cells. During subsequent endosymbiotic evolution, most of mitochondrial original genes have been lost and/or transferred to the nucleus in the eukaryotic cells, so that the mere reminiscing 37 genes are retained in mammalian mitochondria [3, 5]. In this genome, only 13 mitochondrial genes encode its specific proteins as essential subunits of the respiratory chain, whilst the remaining 24 genes encode specific tRNA and rRNA required for its protein translation within the mitochondrial matrix [8, 9]. Such a small complement of mitochondrial genes exists over the entire evolutionary process to strike a balance between the host and its endosymbionts [10, 11]. However, the mitochondrial genetic system only possesses a semiautonomous nature, such that its mtDNA replication, subsequent transcription, and translation are all subjected to the predominant control by several nuclear gene-encoding factors [9, 11–13], e.g., mitochondrial transcription factor A (TFAM), B1 (TFB1M), and B2 (TFB2M) [14–17]. Transcriptional expression of these three factors is regulated by nuclear respiratory factor-1 (NRF-1) [18–20]. Here, NRF-1 is also referred to as *α*-Pal^NRF1^, because it was originally designated *α*-Pal from directly binding to the palindromic consensus site (5′-TGCGCATGCGCA-3′) that is essential for the transcription of *eIF2α* gene [21–23], which controls the general protein translation process and also alternative translation under ER stress. Besides, it is of importance to notice that mitochondrial structure and function, as well as its biogenesis, are also monitored dominantly by those nuclear genome-encoded proteins [17, 24, 25]. Notably, all the other (except the aforementioned 13) subunits of both the mitochondrial respiratory chain and oxidative phosphorylation are transcriptionally controlled by nuclear respiratory factors NRF-1 (i.e., *α*-Pal^NRF1^) and NRF-2 (originally called GA-binding protein, which is thus abbreviated as GABP^NRF2^ but with no any homology with *α*-Pal^NRF1^) binding to distinct consensus sites within these nuclear-encoding respiratory gene promoters [26, 27].

Apart from mitochondria, the eukaryotic endomembrane system including the ER and nucleus has been posited to originate from the bacterial-like out membrane vesicles (OMVs) released by the endosymbiont mitochondrial ancestor within the cytosol of its archaeal host at eukaryote origin [1, 6]. The OMV-based model accounts for (i) the functional homology of the ER and mitochondrial intermembrane space, with dynamic topological exchanges of Ca^2+^ storage and the disulfide relay system required for redox signaling; (ii) the cooperative contributions of the ER and mitochondria to eukaryotic lipid synthesis, as opposed to occurring at the plasma membrane as in all prokaryotes; (iii) the distinction in lipid compositions of between the endomembrane system and plasma membrane, which originated from the fact that bacterial-like lipids replaced the archaeal lipids from the inside in the first place; and that (iv) the ER-continuous nuclear envelope is not homologous to the plasma membrane of putative archaeal host, which is just due to the formation of newly enveloped nucleus from the ER in eukaryotic cells with open mitosis [6]. Recently, functional and proteomic studies have revealed the remarkable complexity of mitochondrial protein machineries with diverse functions, such as protein translocation, oxidative respiration, metabolite transport, protein quality control, and the control of membrane architecture, that also interact with each other in dynamic networks [28]. These protein networks form distinct membrane contact sites, for example, with the ER, which are key for integrity of mitochondria with eukaryotic cellular functions in bioenergetic synthesis, oxidative metabolism, and redox signaling. The ER–mitochondria connections can also, in turn, determine mitochondrial fusion-fission dynamics. Furtherly, the mitochondrial dynamics and inheritance during cell division and development are tightly regulated through evolutionary conserved mechanisms [29], such that they are properly segregated and partitioned as a functional set of their organelles to each of daughter cells during cell division, oogenesis, fertilization, and subsequent development, as well as to ensure the integrity of its mtDNA during genetic selection of functional genomes. Contrarily, defects in the processes lead to relevant cell and tissue pathologies, including cancer and degenerative diseases [29, 30]. However, whether the putative ER-nuclear-mitochondrial (ENUM) communication is involved in mitochondrial regulation remains elusive.

Aside from only 13 mitochondrially encoded *per se* proteins [9], all other thousands of the host nucleus-encoded mitochondrial proteins for its biogenesis, structure, and functions in the cellular respiration, oxidative phosphorylation, energy metabolism, and redox balance are subjected to their temporospatial precision expression [10, 28]. Of note, a considerable number of the nucleus-encoded mitochondrial proteins are transcriptionally regulated by *α*-Pal^NRF1^ and GABP^NRF2^ [26, 27] and also translationally monitored by *α*-Pal^NRF1^-targeted eIF2*α* [21–23]. Particularly, another portion of those nucleus-encoded proteins, that are positioned within and around the outer and inner mitochondrial membranes, are allowed for biosynthesis in proximity to the ribosome-budded ER, because the ER is central to biosynthesis of secretory and membrane proteins, their proper folding and processing into maturation by quality controls [31, 32], before transfer to the mitochondria and anchored within double mitochondrial membranes. The intermembrane space between mitochondrial membranes is evolutionarily homologous to the most oxidizing lumen of the ER, with a lowest GSH/GSSG ratio of 1 : 1 ~ 3 : 1, than those in the relative reducing mitochondrial matrix and nuclear environments (with a higher GSH/GSSG ratio of ~100 : 1) [33–35]. The reducing nucleus is segregated by the ER-connected envelope membranes from the relative oxidizing cytoplasm (GSH/GSSH = 30 : 1 ~ 50 : 1) [33], in which the mitochondria is a major source of reactive oxygen species (ROS) as byproducts during cellular respiration [36]. However, contribution of the ER-derived redox signaling mechanism to the nuclear-to-mitochondrial respiratory and antioxidant transcription networks remains unclear.

Amongst the putative ER-derived redox signaling machineries, the most critical antioxidant arm is mediated by the membrane-bound nuclear factor erythroid-2 p45-related factor 1 (Nrf1, also called Nfe2l1) [37]. The abbreviation is almost identical with nuclear respiratory factor-1 (NRF-1, also called *α*-Pal^NRF1^), leading to the confused interpretation of relevant works, of which the most typical was published by L'Honore et al. (with no precision correction, as compared with its original version online in *Developmental Cell* [38]). As a matter of fact, Nfe2l1 (i.e., Nfe2l1^Nrf1^) is significantly distinctive from *α*-Pal^NRF1^, because the former Nfe2l1^Nrf1^ belongs to the family of cap'n'collar (CNC) basic region-lucine zipper (bZIP) transcription factors that regulate distinct subsets of antioxidant response element (ARE, 5′-TGAC/GnnnGC-3′)-driven cytoprotective genes [37]. This conserved CNC-bZIP family also includes Nfe2l2^Nrf2^ that is negatively regulated by Kelch-like ECH-associated protein 1 (Keap1), which is an adaptor subunit of Cullin 3-based E3 ubiquitin ligase and also a key sensor for oxidative and electrophilic stresses [39]. However, hitherto no literature is available for explaining two distinct, but previously confused, Nrf1 transcription factors, albeit they shared the same abbreviations. Herein, we have identified that both Nfe2l1^Nrf1^ and *α*-Pal^NRF1^ exert synergistic and antagonistic roles in integral regulation of the nuclear to mitochondrial respiratory and antioxidant transcription profiles. Such synergistic contributions of Nfe2l1 and Nfe2l2 to positive regulation of *α*-Pal^NRF1^ and TFAM were validated in *Keap1^−/−^* mouse embryonic fibroblasts (MEFs). By contrast, human *α*-Pal^NRF1^ expression was almost unaltered by *hNfe2l1α^−/−^* or *hNfe2l2^-/-ΔTA^*, albeit its target TFAM was activated by Nfe2l1, along with antagonism against Nfe2l2 in HepG2 cells. Conversely, almost all of mouse Nfe2l1, Nfe2l2, and cotarget genes were downexpressed to certain extents in *α-Pal^NRF1+/-^* MEFs. Further investigation revealed that human Nfe2l1, Nfe2l2, and relevant reporter genes were downregulated by an ectopic *α*-Pal^NRF1^ expression, but this inhibitory effect was reversed by silencing *α*-Pal^NRF1^. Moreover, Pitx2 (pituitary homeobox 2) was identified as a direct upstream regulator of Nfe2l1 and TFAM, besides *α*-Pal^NRF1^. Collectively, these across-talks between Nfe2l1, Nfe2l2, and *α*-Pal^NRF1^, together with Pitx2, are integrated through multiple extranuclear (e.g., ER-derived) signaling to the nuclear-to-mitochondrial communication for their mitochondrial target TFAM, so as to finely tune distinct cellular oxidative respiratory and antioxidant gene transcription networks responsible for maintaining the robust redox homeostasis and organelle integrity, albeit they differ between the mouse and human.

## 2. Results

### 2.1. Knockout of Nfe2l1^Nrf1^ in MEFs Leads to Substantial Decreases of Nfe2l2, *α*-Pal^NRF1^, TFAM, and Other Target Genes

**Figure 1:**
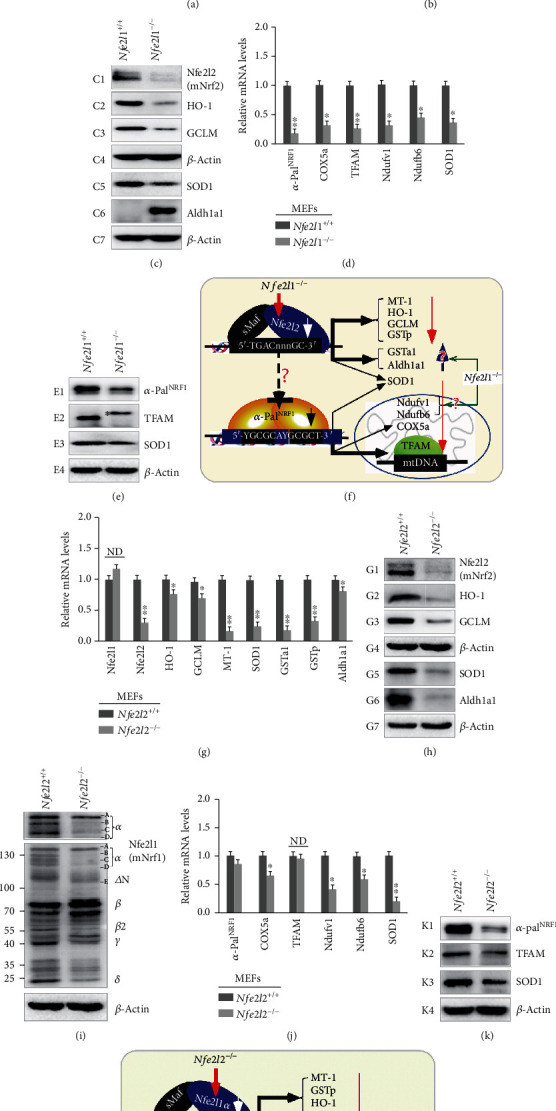
Distinct effects of *Nfe2l1^−/−^*and *Nfe2l2^−/−^* on the basal expression of mouse *α-Pal^NRF1^*, *TFAM*, and relevant genes in MEFs. (a) The mRNA levels of *Nfe2l1* and *Nfe2l2*, as well as indicated antioxidant genes, were determined by real-time qPCR of *Nfe2l1^−/−^* and *Nfe2l1^+/+^* MEFs. The data are shown as mean ± SEM (*n* = 3 × 3) with significant decreases (^∗^*p* < 0.01, ^∗∗^*p* < 0.001) or increases ($, *p* < 0.01; $$, *p* < 0.001). (b) Distinct Nfe2l1 isoforms, along with a loading control *β*-actin, were also examined by Western blotting of *Nfe2l1^−/−^* and *Nfe2l1^+/+^* MEFs. (c) Changes in basal protein levels of Nfe2l2 and antioxidant enzymes HO-1, GCLM, SOD1, and Aldh1a1 between *Nfe2l1^−/−^* and *Nfe2l1^+/+^* MEFs were also observed. (d) Alterations in the basal mRNA expression levels of *α-pal^NRF1^*, *COX5a*, *TFAM*, *Ndufv1*, *Ndufb6*, and *SOD1* were unraveled by real-time qPCR of *Nfe2l1^−/−^* and *Nfe2l1^+/+^* MEFs. The results are shown as mean ± SEM (*n* = 3 × 3) with significant decreases (^∗^*p* < 0.01, ^∗∗^*p* < 0.001). (e) Altered proteins of *α*-Pal^NRF1^, TFAM, and SOD1 between *Nfe2l1^−/−^* and *Nfe2l1^+/+^* MEFs were visualized by Western blotting. (f) A model is proposed to present possible effects of *Nfe2l1^−/−^* on the basal expression of *Nfe2l2*, *α-Pal^NRF1^*, *TFAM*, and relevant genes in MEFs. (g) Basal mRNA levels of *Nfe2l1* and Nfe2l2, as well as indicated antioxidant genes, in between *Nfe2l2^−/−^* and *Nfe2l2^+/+^* MEFs were also comparatively analyzed. The results are shown as mean ± SEM (*n* = 3 × 3) with significant decreases (^∗^*p* < 0.01, ^∗∗^*p* < 0.001) or ND (no statistical difference). (h) Distinct abundances of Nfe2l2 and antioxidant enzymes in between *Nfe2l2^−/−^* and *Nfe2l2^+/+^* MEFs were determined by Western blotting. (i) Altered abundances of distinct Nfe2l1 isoforms in *Nfe2l2^−/−^* from *Nfe2l2^+/+^* MEFs were found. (j) The mRNA expression levels of *α-Pal^NRF1^*, *COX5a*, *TFAM*, *Ndufv1*, *Ndufb6*, and *SOD1* were analyzed by comparing real-time qPCR data from *Nfe2l2^−/−^* and *Nfe2l2^+/+^* MEFs. The results are shown as mean ± SEM (*n* = 3 × 3) with significant decreases (^∗^*p* < 0.01, ^∗∗^*p* < 0.001) or ND (no statistical difference). (k) Abundances of *α*-Pal^NRF1^, TFAM, and SOD1 were compared by immunoblotting of *Nfe2l2^−/−^* with *Nfe2l2^+/+^* MEFs. (l) Another model is proposed to present potential influence of *Nfe2l2^−/−^* on *Nfe2l1*, *α-Pal^NRF1^*, *TFAM*, and relevant genes (and their proteins) in MEFs.

Herein, whether knockout of *Nfe2l1* has an effect on the constitutive expression of Nfe2l2 and target genes in MEFs was firstly examined by real-time quantitative PCR (RT-qPCR) and Western blotting. As anticipated, loss of *Nfe2l1* in mice resulted in substantial decreases in both mRNA and protein levels of Nfe2l2 expressed constitutively in *Nfe2l1^−/−^* MEFs (Figures 1(a)–1(c)). Similar decreases in the basal expression of five of the examined *ARE*-driven genes *HO-1* (heme oxygenase 1, HMOX1), *GCLM* (glutamate-cysteine ligase modifier subunit), *MT-1* (metallothionein 1), *GSTp* (glutathione S-transferase pi 1), and *SOD1* (superoxide dismutase 1) were determined in *Nfe2l1^−/−^* MEFs (Figure 1(a)). By sharp contrast, significant increases in the basal expression of *GSTa1* (glutathione S-transferase, alpha 1 (Ya)) and *Aldh1a1* (aldehyde dehydrogenase family 1, A1) were also obtained from *Nfe2l1^−/−^* in MEFs (Figure 1(a)), although they were identified as Nfe2l2-target genes [40, 41]. Western blotting of *Nfe2l1^−/−^* MEFs revealed obvious decreases in protein abundances of HO-1, GCLM, and SOD1, as accompanied by a striking increase in Aldh1a1 protein levels (Figure 1(c)). In addition, it should also be noted that Nfe2l1*α* and its derivates were completely deleted in *Nfe2l1^−/−^* MEFs, but the residual shorter isoforms *Δ*N, *β*, *γ*, and *δ* were detected by Western blotting with the antibodies against amino acids 291-741 of Nfe2l1^Nrf1^ (Figure 1(b)) [42]. Collectively, these indicate bidirectional contributions of Nfe2l1 to positive and negative regulation of ARE-driven genes by Nfe2l2 or Nfe2l1*α*.

Further RT-qPCR analysis of *Nfe2l1^−/−^* MEFs revealed that loss of Nfe2l1*α* also led to massive decreases in mRNA expression levels of *α-Pal^NRF1^* and its target genes *TFAM*, *COX5a* (cytochrome c oxidase subunit 5A), *Ndufv1* (NADH:ubiquinone oxidoreductase core subunit V1), and *Ndufb6* (NADH:ubiquinone oxidoreductase subunit B6), besides *SOD1* (Figure 1(d)). Substantial decreases in protein abundances of *α*-Pal^NRF1^, TFAM, and SOD1 were visualized by Western blotting of *Nfe2l1^−/−^* MEFs (Figure 1(e)). These demonstrate that Nfe2l1*α* (and its derivates) is required for expression of *α*-Pal^NRF1^ and its downstream targets, including TFAM (Figure 1(f)).

### 2.2. Knockout of Nfe2l2^Nrf2^ in MEFs Results in Substantial Decreases of *α*-Pal^NRF1^, TFAM, and Other Target Genes

As shown in Figure 1(g), the basal mRNA expression of *Nfe2l1* in MEFs was almost unaffected by knockout of *Nfe2l2^−/−^*, albeit all 7 of the examined Nfe2l2-targeted genes *HO-1*, *GCLM*, *MT-1*, *SOD1*, *GSTa1*, *GSTp*, and *Aldh1a1* were downregulated by loss of *Nfe2l2* to different lower extents, when compared to their equivalent levels measured in wild-type (*Nfe2l2^+/+^*) cells. The latter notion is also corroborated by evidence showing significant decreases in the constitutive protein expression of HO-1, GCLM, SOD1, and Aldh1a1 (Figure 1(h)). Intriguingly, the full-length Nfe2l1*α* abundance was only marginally reduced in *Nfe2l2^−/−^* MEFs, but its processed isoforms of protein-B to -D appeared to be largely abolished by *Nfe2l2^−/−^*, which was also accompanied by a rather remarkable increased abundance of Nfe2l1*β* (Figure 1(i)). These findings suggest that the putative proteolytic processing of Nfe2l1*α* to yield distinct isoforms may be influenced by Nfe2l2.

Comparative analysis of *Nfe2l2^−/−^* and wild-type MEFs unraveled almost no changes in mRNA expression levels of *α-Pal^NRF1^* and *TFAM* (Figure 1(j)). However, both protein levels of *α*-Pal^NRF1^ and TFAM were markedly downregulated by loss of *Nfe2l2* (Figure 1(k)), implying that the stabilization of *α*-Pal^NRF1^ and TFAM may be monitored in the presence of Nfe2l2 (Figure 1(l)). Intriguingly, the mRNA expression of *COX5a*, *Ndufv1*, and *Ndufb6*, besides *SOD1*, were downregulated to varying degrees in *Nfe2l2^−/−^* MEFs (Figure 1(j)). This suggests that Nfe2l2 is likely required for controlling the basal expression of these three mitochondrial respiratory genes regulated through an *α*-Pal^NRF1^-independent mechanism (Figure 1(l)).

### 2.3. Significant Increases of Nfe2l1, Nfe2l2, *α*-Pal^NRF1^, TFAM, and Other Target Genes in Keap1^−/−^MEFs

**Figure 2:**
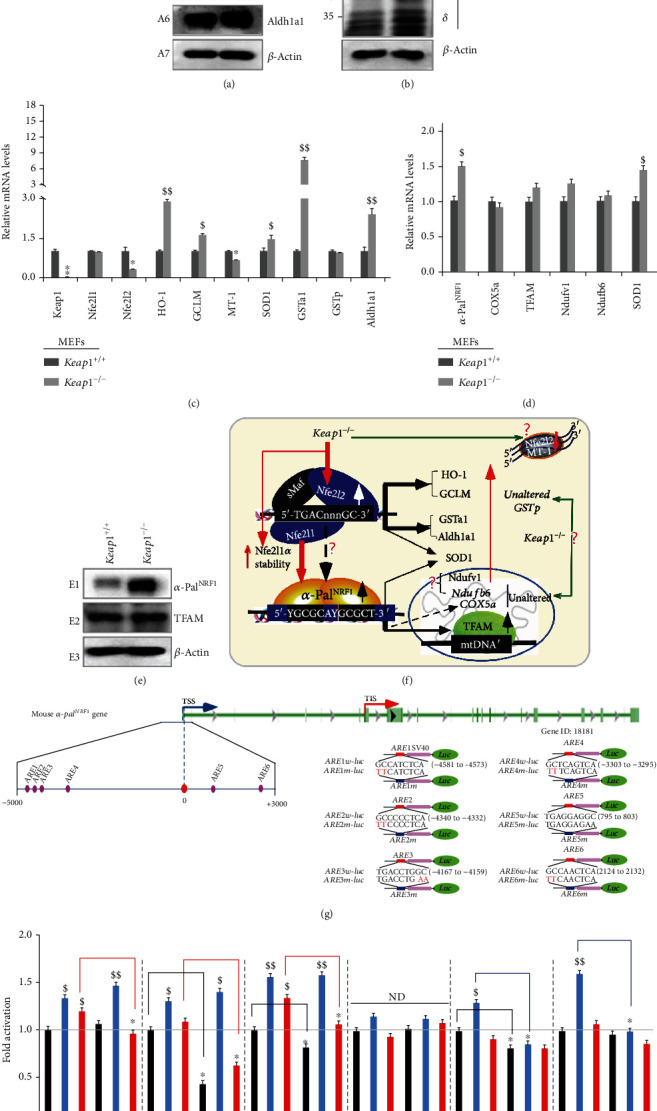
Distinct roles of key redox control genes in the basal expression of mouse *α-Pal^NRF1^*, *TFAM*, and relevant genes. (a) Distinct protein levels of keap1 and Nfe2l2, as well as indicated antioxidant enzymes, in between *Keap1^−/−^* and *Keap1^+/+^* MEFs were visualized by Western blotting. (b) Altered abundances of distinct Nfe2l1 isoforms between *Keap1^−/−^* and *Keap1^+/+^* MEFs were observed. (c) Basal mRNA levels of *keap1*, *Nfe2l1*, and *Nfe2l2*, as well as indicated antioxidant genes, were determined by real-time qPCR of *Keap1^−/−^* and *Keap1^+/+^* MEFs. The data are shown as mean ± SEM (*n* = 3 × 3) with significant decreases (^∗^*p* < 0.01, ^∗∗^*p* < 0.001) or significant increases ($, *p* < 0.01; $$, *p* < 0.001). (d) Basal mRNA levels of *α-Pal^NRF1^*, *COX5a*, *TFAM*, *Ndufv1*, *Ndufb6*, and *SOD1* were also determined as described above. (e) Altered protein levels of *α*-Pal^NRF1^ and TFAM were revealed by Western blotting of *Keap1^−/−^* and *Keap1^+/+^* MEFs. (f) A model is proposed to present effects of *Keap1^−/−^* on *Nfe2l1*, *Nfe2l2*, *α-Pal^NRF1^*, *TFAM*, and related genes (and their proteins) in MEFs. (g) Within the promoter region of mouse *α-Pal^NRF1^* gene, the putative *ARE* sites (each with the core sequence 5′-TGAC/GnnnGC-3′) were marked (as purple dots, *left panel*). Six distinct *ARE*-driven reporters (i.e., *ARE1* to *ARE6*-*luc*) and their respective mutants were constructed into the pGL3-Promoter vector (*right panel*). (h) Each pair of indicated *ARE-luc* and mutants was cotransfected with the internal control pRL-TK, together with each of expression constructs for mouse Nfe2l1, Nfe2l2, or empty pcDNA3.1 into RL34 cells for 8 h, before being allowed for 24 h recovery. Subsequently, distinct *ARE*-driven luciferase activity was measured. The resultant data are shown as mean ± SEM (*n* = 3 × 3) with significant increases ($, *p* < 0.01; $$, *p* < 0.001) or decreases (^∗^*p* < 0.01). ND: no statistical difference.

Knockout of the Nfe2l2-inhibitor Keap1 caused significant increases in protein abundances of Nfe2l2 and its downstream targets HO-1, GCLM, Aldh1a1, and SOD1 in *Keap1^−/−^* MEFs (Figure 2(a)), as consistent with the previous work [43]. Of note, basal abundances of Nfe2l1-*α*, -*Δ*N, -*γ*, and -*δ*, but not -*β*, isoforms were incremented in *Keap1^−/−^* MEFs (Figure 2(b)), albeit its mRNA expression was unaltered by loss of *Keap1* (Figure 2(c)). However, it is, to our surprise, that a substantial decrease of *Nfe2l2* at its basal mRNA expression levels was found in *Keap1^−/−^* MEFs (Figure 2(c)). These observations demonstrate that increased abundances of Nfe2l2, and also possibly Nfe2l1, proteins are negatively monitored by a Keap1-mediated degradation pathway. Such coactivation of Nfe2l2 and Nfe2l1 by *Keap1^−/−^* led to distinct extents of upregulation of ARE-driven genes *HO-1*, *GSTa1*, *Aldh1a1*, *GCLM*, and *SOD1* but not *GSTp* or *MT-1* (Figure 2(c)). In fact, *GSTp* was almost unaffected, whilst *MT-1* was marginally suppressed, by *Keap1^−/−^-*led coactivation of Nfe2l2 and Nfe2l1 (Figure 2(c)), even though both*GSTp* and *MT-1*mRNA expression levels were substantially downregulated in either *Nfe2l1^−/−^* or *Nfe2l2^−/−^* MEFs (Figure 1(a) and 1(g)), no matter whether Nfe2l1*β* was decreased in *Nfe2l1^−/−^* (Figure 1(b)) or conversely increased in *Nfe2l2^−/−^* MEFs (Figure 1(h)). Thereby, it is inferable that both *GSTp* and *MT-1* genes (and possibly *Nfe2l2 per se*) are also likely subjected to putative dominant negative regulation by, at least, a not-yet-identified transcription factor competitively against the positive effects of Nfe2l2 and Nfe2l1 in *Keap1^−/−^* MEFs.

By contrast, further examination of *Keap1^−/−^* MEFs uncovered remarkable increases of both *α*-Pal^NRF1^ and TFAM in their mRNA and protein expression levels (Figure 2(d) and 2(e)). This finding demonstrates upregulation of both *α*-Pal^NRF1^ and TFAM by *Keap1^−/−^-*led coactivation of Nfe2l2 and Nfe2l1. However, *Ndufv1* and *Ndufb6* (both encoding two distinct subunits of the mitochondrial respiratory chain complex *Ι*) were only slightly promoted, whilst the expression of *COX5a* was roughly unaltered, in *Keap1^−/−^* MEFs (Figure 2(d)). This result implies a putative negative transcription factor competitively against the positive regulatory effects of *α*-Pal^NRF1^ on the transcriptional expression of its three mitochondrially-targeted genes *Ndufv1*, *Ndufb6*, and *COX5a* encoded in the nucleus of *Keap1^−/−^* MEFs (Figure 2(f)).

### 2.4. Induction of ARE-Driven *α*-Pal^NRF1^-Luciferase Reporter Genes Mediated by Ectopic Nfe2l1 and/or Nfe2l2

To further determine the transcriptional regulation of *α*-Pal^NRF1^ by Nfe2l1 and Nfe2l2, we herein created 6 pairs of wild-type luciferase reporters driven by the indicated *ARE* consensus sites from mouse *α-Pal^NRF1^*promoter and their corresponding mutants (as shown in Figure 2(g)). Amongst them, the basal activity of *ARE2*, *ARE3*, and *ARE5* was obviously prevented by their respective mutants (Figure 2(h)). This implies putative regulation of these three reporters by endogenous *ARE*-binding factors Nfe2l1 and/or Nfe2l2 alone or both together. Further cotransfection of RL34 cells with an Nfe2l1^Nrf1^ expression construct revealed that the transcriptional expression of all other 5 *ARE*-, except *ARE4*-, driven *luciferase* reporters was activated by Nfe2l1 (Figure 2(h)). Such Nfe2l1-mediated activity of only ARE5 and ARE6 situated within the first exon was suppressed by their mutants, but other three *ARE1*, *ARE2*, and *ARE3* activity mediated by Nfe2l1 was largely unaffected by their mutants (Figure 2(h)). By contrast, transcriptional activity of *ARE1-*, *ARE2*-, and *ARE3-luc* reporters was not only activated by Nfe2l2 but also reduced by their respective mutants to relative lower degrees. Together, these indicate that *ARE1*, *ARE2*, and *ARE3* are Nfe2l2-specific regulatory sites within the mouse *α-Pal^NRF1^* promoter region, whilst *ARE5* and *ARE6* are Nfe2l1-specific regulatory sites within the 5′-noncoding exon one-encoded region of *α-Pal^NRF1^*. In addition, *ARE1-*, *ARE2*-, and *ARE3-*mutant reporters were also activated by Nfe2l1 but not by Nfe2l2. This suggests a possibility that the three ARE mutants from the *α-Pal^NRF1^* promoter region could also serve as certain artificial consensus sites recruiting the putative Nfe2l1-downstream transcription factors. Thus, it is inferable that upregulation of *ARE1*, *ARE2*, and *ARE3* activity to mediate the *α-Pal^NRF^* expression may also be mediated by Nfe2l1 directly and/or indirectly through its target downstream factors.

### 2.5. Downregulation of Mouse Nfe2l1, Nfe2l2, and ARE-Driven Genes by Heterogeneous Deletion of *α*-Pal^NRF1^ in MEFs

**Figure 3:**
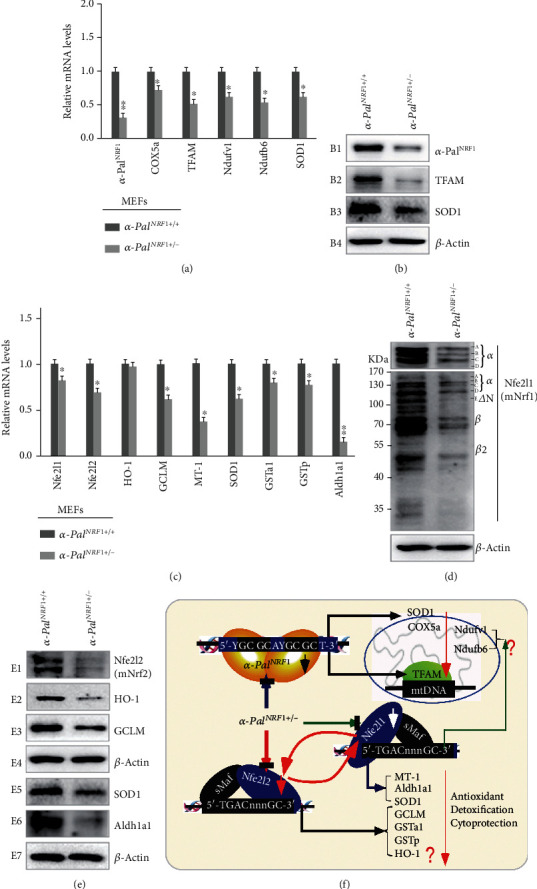
Distinct effects of mouse *α-pal^NRF1+/-^* on the expression of *Nfe2l1*, *Nfe2l2*, *TFAM*, and related genes. (a) Altered mRNA levels of *α-pal^NRF1^*, *COX5a*, *TFAM*, *Ndufv1*, *Ndufb6*, and *SOD1* in *α-pal^NRF1+/-^* MEFs were compared with their equivalents measured from wild-type (*α-pal^NRF1+/+^*) cells. The data are shown as mean ± SEM (*n* = 3 × 3) with significant decreases (^∗^*p* < 0.01, ^∗∗^*p* < 0.001). (b) Significant changes in *α*-Pal^NRF1^, TFAM, and SOD1 proteins were detected by Western blotting of *α-pal^NRF1+/-^* and *α-pal^NRF1+/+^* MEFs. (c) Changes in basal mRNA levels of *Nfe2l1* and *Nfe2l2*, as well as indicated antioxidant genes, were determined by real-time qPCR of *α-pal^NRF1+/-^* MEFs, when compared with *α-pal^NRF1+/+^* MEFs. The results are shown as mean ± SEM (*n* = 3 × 3) with significant decreases (^∗^*p* < 0.01, ^∗∗^*p* < 0.001). (d) Altered abundances of distinct Nfe2l1 isoforms in between *α-pal^NRF1+/-^* and *α-pal^NRF1+/+^* MEFs were also visualized. (e) Altered protein levels of Nfe2l2 and the indicated antioxidant enzymes, such as HO-1, GCLM, SOD1, and Aldh1a1, were further unraveled by Western blotting of *α-pal^NRF1+/-^* and *α-pal^NRF1+/+^* MEFs. (f) A model is proposed to explain distinct effects of *α-pal^NRF1+/-^* on the nuclear-to-mitochondrial respiratory and antioxidant genes in MEFs.

In turn, to explore whether *α*-Pal^NRF1^ contributes to expression of *Nfe2l1*, *Nfe2l2*, and *ARE-*driven genes, we employed the CRISPR-Cas9-mediated genome-editing of wild-type MEFs to create a heterogenous mutant cell line called *α-Pal^NRF1+/-^* (but the homogenous mutant*α-Pal^NRF1-/-^*cells could not be acquired due to its lethality). This cell line was further confirmed to be true by its DNA sequencing and RT-qPCR (Figures 3(a) and S1). Accordingly, basal expression levels of those *α*-Pal^NRF1^-target genes *TFAM*, *Ndufv1*, *Ndufb6*, *COX5a*, and *SOD1* were markedly decreased, as *α*-Pal^NRF1^ was deleted heterogeneously in *α-Pal^NRF1+/-^* MEFs (Figures 3(a) and 3(b)). Interestingly, further RT-qPCR analysis revealed that basal expression levels of *Nfe2l1*, *Nfe2l2*, and *ARE-*driven genes *GCLM*, *MT-1*, *Aldh1a1*, *GSTa1*, and *GSTp1*, besides *SOD1*, were evidently reduced by *α-Pal^NRF1+/-^* to different lower extents, when compared with those equivalents of *α-Pal^NRF1+/+^* cells (Figure 3(c)). Western blotting of *α-Pal^NRF1+/-^* cells unraveled substantial decreases in distinct isoforms of mouse *Nfe2l1* and *Nfe2l2* proteins (Figures 3(d) and 3(e)). Consistently, abundances of HO-1, GCLM, Aldh1a1, and SOD1 proteins were strikingly decreased in *α-Pal^NRF1+/-^* MEFs (Figure 3(e)). Collectively, these findings demonstrate a putative contribution of *α*-Pal^NRF1^ (and its signaling) to constructive expression of *Nfe2l1*, *Nfe2l2*, and cognate *ARE-*driven target genes (Figure 3(f)). In addition to their transcriptional expression, their posttranslational processes may also be influenced by *α*-Pal^NRF1^ signaling. This is based on the observations that significantly decreases in abundances of Nfe2l1 and HO-1 proteins were, rather, accompanied by less or no changes in their mRNA expression levels, respectively, by comparison of *α-Pal^NRF1+/-^* with wild-type (*α-Pal^NRF1+/+^*) MEFs (Figures 3(c)–3(e)).

### 2.6. No Alterations of *α*-Pal^NRF1^ in *hNfe2l1α*^−/−^ or *hNfe2l2*^-/-*Δ*TA^ Are Accompanied by Opposing Changes of TFAM

**Figure 4:**
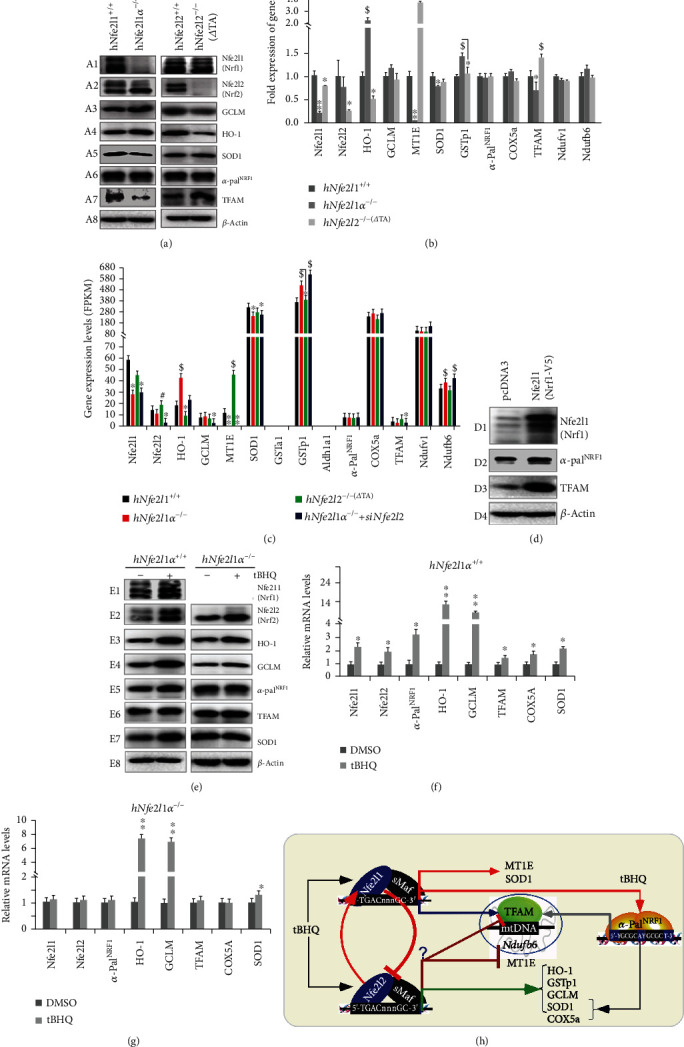
Distinct changes of human *α*-Pal^NRF1^ and TFAM in HepG2-derived *hNfe2l1α^−/−^* and *hNfe2l2^−/−^* cell lines. (a) Distinct protein levels of human Nfe2l1, Nfe2l2, *α*-Pal^NRF1^, and TFAM as well as other relevant proteins were determined by Western blotting of *hNfe2l1α^−/−^*, *hNfe2l2^−/−^*, and wild-type HepG2 cells. (b) Basal mRNA expression levels of *Nfe2l1*, *Nfe2l2*, *α-Pal^NRF1^*, *TFAM*, and other indicated genes were examined by real-time qPCR of *hNfe2l1α^−/−^*, *hNfe2l2^−/−^*, and wild-type HepG2 cells. The resultant data are shown as mean ± SEM (*n* = 3 × 3) with significant decreases (^∗^*p* < 0.01, ^∗∗^*p* < 0.001) or significant increases ($, *p* < 0.01; $$, *p* < 0.001). (c) The FPKM (Reads Per Kilobase per Million mapped reads) value of *Nfe2l1*, *Nfe2l2*, *α-Pal^NRF1^*, *TFAM*, and other indicated genes were obtained by RNA-sequencing of *hNfe2l1α^−/−^*, *hNfe2l2^−/−^*, *hNfe2l1α^−/−^+siNfe2l2*, and *hNfe2l1/2^+/+^*. (d) Different protein levels of Nfe2l1, *α*-Pal^NRF1^, and TFAM were examined in HepG2 cells that had been transfected with an hNfe2l1 expression construct or empty pcDNA3.1. (e) Distinct inducible alterations in abundances of Nfe2l1, Nfe2l2, *α*-Pal^NRF1^, TFAM, HO-1, GCLM, and SOD1 were determined by Western blotting of *hNfe2l1α^+/+^* or *hNfe2l1α^−/−^* that had been or not been treated with 50*μ*mol/L tBHQ. (f, g) Distinct inducible mRNA levels of *Nfe2l1*, *Nfe2l2*, *α-Pal^NRF1^*, *HO-1*, *GCLM*, *TFAM*, *COX5a*, and *SOD1* were revealed by real-time qPCR of between tBHQ-stimulated lines of *hNfe2l1α^+/+^* cells (f) and *hNfe2l1α^−/−^* cells (g). The resultant data are shown as mean ± SEM (*n* = 3 × 3) with significant increases (^∗^*p* < 0.01, ^∗∗^*p* < 0.001). (h) A model is assumed to present cross-talks between human Nfe2l1 and Nfe2l2, along with distinct effects on human *α*-Pal^NRF1^, TFAM, and other gene expression, particularly upon stimulation by tBHQ.

Next, we examined the inter-regulatory effects of between human Nfe2l1 and Nfe2l2 on *α*-Pal^NRF1^, TFAM, and other target genes. Unexpectedly, almost no changes in protein and mRNA expression levels of *α*-Pal^NRF1^ were determined in either *hNfe2l1α^−/−^* or *hNfe2l2^-/-ΔTA^* cells (both lines were established from human HepG2 cells by Qiu et al. [44]), when compared with those equivalents obtained from wild-type cells (Figures 4 A6 and 4(b)). Accordingly, no or less alterations in the basal expression of *Ndufv1*, *Ndufb6*, and *COX5a* by *hNfe2l1α^−/−^* or *hNfe2l2^-/-ΔTA^* were observed (Figure 4(b)). However, it is intriguing that *α*-Pal^NRF1^-target TFAM at its protein and mRNA levels was strikingly downregulated in *hNfe2l1α^−/−^* cells (albeit with hyper-expression of Nfe2l2), but rather significantly upregulated in *hNfe2l2^-/-ΔTA^* cells, by comparison with their wild-type controls (Figures 4 A7 and 4(b)). These observations were further substantiated by transcriptomic sequencing (Figure 4(c)). Collectively, these results indicate that both Nfe2l1 and Nfe2l2 contributes, respectively, to the putative positive and negative regulation of the human TFAM expression, possibly through a mechanism independent of *α*-Pal^NRF1^. This notion is further supported by the evidence showing that a substantial increase in protein abundances of TFAM, rather than *α*-Pal^NRF1^, was resulted from overexpression of ectopic Nfe2l1 (i.e., Nrf1-V5, as shown in Figure 4, cf. D3 with D4).

In addition, the inter-regulatory effects of between human Nfe2l1 and Nfe2l2 on the ARE-driven genes were also validated, as consistent with our previous report [44]. Of note, knockout of *hNfe2l1α^−/−^* caused a marked increase in human Nfe2l2 protein abundances but not in its mRNA expression (Figures 4 A2, 4(b), and 4(c)). In turn, *hNfe2l2^-/-ΔTA^* cells gave rise to an obvious reduction of post-synthetically processed Nfe2l1 isoforms and its mRNA levels (Figures 4 A1, 4(b), and 4(c)).

Western blotting of *hNfe2l1α^−/−^* revealed that massive increases of both GCLM and HO-1 proteins were coincided with hyperactive Nfe2l2 (Figure 4, *A3* and *A4*). RT-qPCR analysis unraveled that the basal mRNA expression of *HO-1* was substantially upregulated in *hNfe2l1α^−/−^* cells but also significantly downregulated in *hNfe2l2^-/-ΔTA^* cells (Figure 4(b)), whereas the *GCLM* mRNA expression was roughly unaltered in these two cell lines, when compared to wild-type controls. Further transcriptomic sequencing unveiled that the basal *GCLM* expression was only repressed by *hNfe2l1α^−/−^+siNrf2* (Figure 4(c)), implying that it is coregulated by both Nfe2l1 and Nfe2l2. Furthermore, *GSTp1* was upexpressed in *hNfe2l1α^−/−^* cells and also further incremented in *hNfe2l1α^−/−^+siNrf2* cells but rather unaltered in *hNfe2l2^-/-ΔTA^* cells, when compared to wild-type controls (Figures 4(b) and 4(c)). By contrast, the basal mRNA expression of *MT1E* (encoding metallothionein 1E) was almost completely abolished in *hNfe2l1α^−/−^* or *hNfe2l1α^−/−^+siNfe2l2* cells but also enormously augmented in *hNfe2l2^-/-ΔTA^* cells (Figures 4(b) and 4(c)). Overall, these demonstrate that human Nfe2l1 and Nfe2l2 contribute to the positive and negative regulation of *MT1E*, respectively, on which Nfe2l1 exerts its dominant effect, but both *GSTp1* and *HO-1* expression is Nfe2l2-dependent. However, the SOD1 expression is Nfe2l1*α*-dependent, because its protein and mRNA levels were reduced in either *hNfe2l1α^−/−^* or *hNfe2l1α^−/−^+siNfe2l2* cells but largely unaffected in *hNfe2l2^-/-ΔTA^* cells (Figures 4 A5, 4(b), and 4(c)).

### 2.7. Induction of Both *α*-Pal^NRF1^ and TFAM by *tert*-Butylhydroquinone (tBHQ) Is Completely Abolished in *hNfe2l1α*^−/−^ Cells

Here, we determined effects of *hNfe2l1α^−/−^* on tBHQ-stimulated expression of *α*-Pal^NRF1^ and TFAM. As anticipated, abundances of *α*-Pal^NRF1^, TFAM, and SOD1 proteins were evidently increased following 50 *μ*mol/L tBHQ treatment of wild-type *hNfe2l1α^+/+^* cells, when compared with the vehicle DMSO-treated controls (Figure 4, *E5* and *E6*). However, similar increases of *α*-Pal^NRF1^, TFAM, and SOD1 were not observed in *hNfe2l1α^−/−^* cells, although hyperactive Nfe2l2 was further incremented by tBHQ (*cf. E2 with E5-E7*). Subsequently, RT-qPCR analysis revealed that mRNA expression levels of *α-Pal^NRF1^*, *TFAM*, *COX5a*, and *SOD1* were induced, to greater or less extents (Figure 4(f)), by tBHQ stimulation of Nfe2l1, but not Nfe2l2, in *hNfe2l1α^+/+^* cells (Figures 4(e) and 4(f)). This is based on the fact that tBHQ-inducible increases of *α-Pal^NRF1^*, *TFAM*, and *COX5a*, rather than *SOD1*, appeared to be completely abolished in *hNfe2l1α^−/−^* cells, albeit Nfe2l2 was hyperexpressed by tBHQ (Figure 4(g)). Altogether, these demonstrate transcriptional regulation of *α-Pal^NRF1^*, *TFAM*, and *COX5a* dominantly by tBHQ-inducible Nfe2l1 but not Nfe2l2, whilst the *SOD1* expression is coregulated by both CNC-bZIP factors (Figure 4(h)). In addition, tBHQ treatment of *hNfe2l1α^+/+^* cells led to significant increases in protein and mRNA levels of HO-1 and GCLM (Figures 4 E3, E4, and 4(f)). Similar induction of HO-1 and GCLM by tBHQ was obtained in *hNfe2l1α^−/−^* cells (Figures 4 E3, E4, and 4(g)). Thus, it is inferable that Nfe2l2 makes a major contribute to induction of the HO-1 and GCLM expression by tBHQ (Figure 4(h)).

### 2.8. Another Contribution of *α*-Pal^NRF1^ to Transrepression of Human Nfe2l1, Nfe2l2, and ARE-Driven Genes

**Figure 5:**
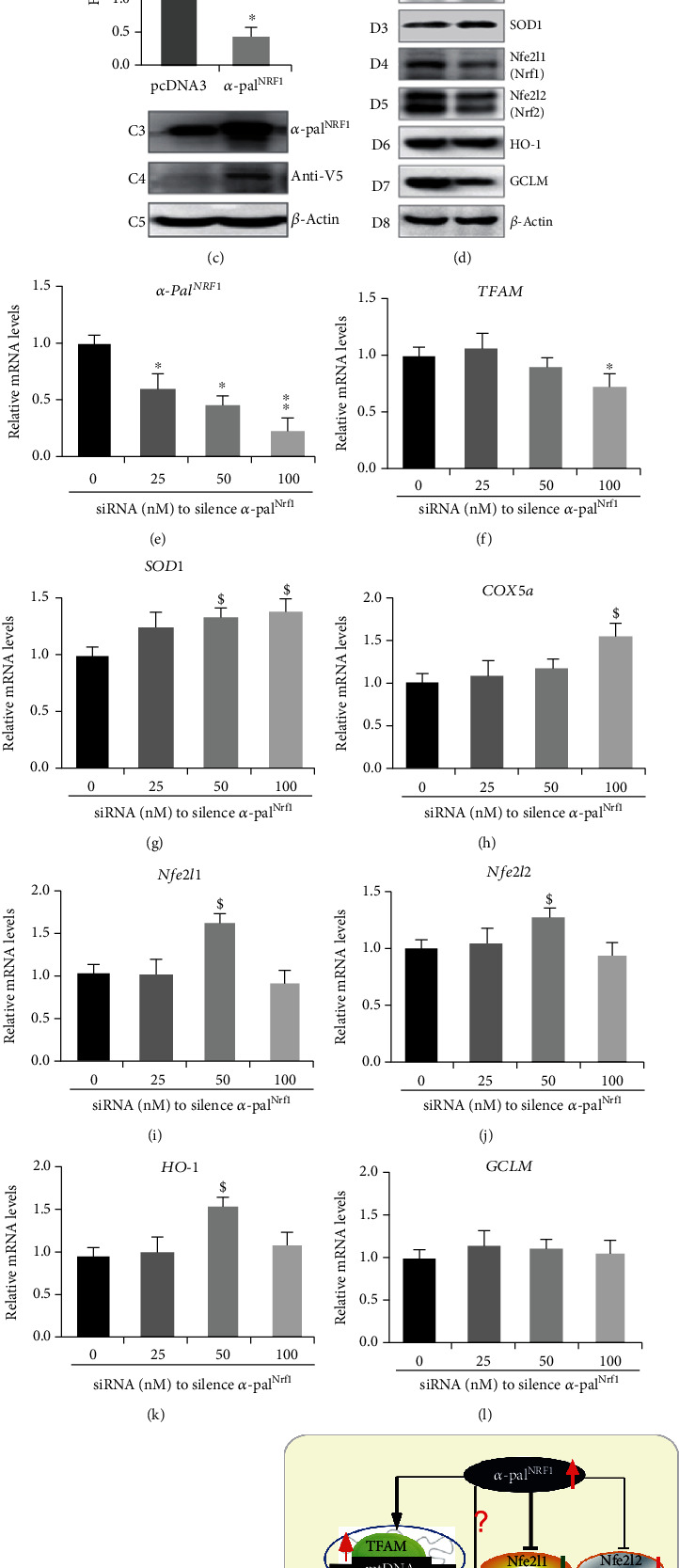
A negative effect of *α*-Pal^NRF1^ on *Nfe2l1*, *Nfe2l2*, and other antioxidant genes in HepG2 cells. (a) HepG2 cells were cotransfected for 8 h with *mNfe2l1-luc* reporter and the pRL-TK control, along with an expression construct for mouse *α*-pal^NRF1^ or an empty pcDNA3.1, and then allowed for a 24 h recovery before the luciferase activity was measured (*A1*). Total cell lysates were also subjected to identification by Western blotting with distinct antibodies against *α*-Pal^NRF1^ (*A2*) or V5 tag (*A3*). (b) HepG2 cells were cotransfected for 8 h with *mNfe2l1-luc* (*B1*) *or hNfe2l1-luc* (*B2*), plus pRL-TK, and then treated with 50 *μ*mol/L tBHQ or the DMSO vehicle for 24 h, before being allowed for additional 24 h recovery. Subsequently, these samples were subjected to dual luciferase assays. The results are shown as mean ± SEM (*n* = 3 × 3) with significant increases ($, *p* < 0.01). (c) HepG2 cells were cotransfected for 8 h with *hNfe2l1-luc* (*C1*) or *ARE×6-luc* (*C2*) together with pRL-TK, plus a human *α*-Pal^NRF1^ expression construct or an empty pcDNA3.1 and then allowed for 24 h recovery from cotransfection, before the reporter activity was measured. The data are shown as mean ± SEM (*n* = 3 × 3) with significant decreases (^∗^*p* < 0.01). Total cell lysates were also subjected to characterization by Western blotting with distinct antibodies against *α*-Pal^NRF1^ (*C3*) or V5 tag (*C4*). (d) Distinct protein levels of *α*-Pal^NRF1^, TFAM, SOD1, Nfe2l1, Nfe2l2, HO-1, and GCLM were determined by Western blotting of HepG2 cells that had been transfected with *α*-Pal^NRF1^ expression plasmid or an empty pcDNA3 vector. (e–l) Distinct changes in mRNA levels of *α-Pal^NRF1^* (e), *TFAM* (f), *SOD1* (g), *COX5a* (h), Nfe2l1 (i), Nfe2l2 (j), HO-1 (k), and GCLM (l) were analyzed by real-time qPCR of HepG2 cells that had been transfected with 0, 25, 50, and 100 nM of siRNA against *α-Pal^NRF1^*. The resulting data are shown as mean ± SEM (*n* = 3 × 3) with significant decreases (^∗^*p* < 0.01, ^∗∗^*p* < 0.001) or increases ($, *p* < 0.01). (m) Such siRNA-transfected cell lysates were subjected to Western blotting analysis of distinct protein abundances as indicated. (n) A model is proposed to present disparate effects of *α*-pal^NRF1^ overexpression or its knockdown on the nuclear-to-mitochondrial respiratory and antioxidant genes in HepG2 cells.

To clarify putative contributions of *α*-Pal^NRF1^ to the transcriptional expression of *Nfe2l1*, we herein made two luciferase reporters driven by mouse and human *Nfe2l1* gene promoter regions, called *mNfe2l1-luc* and *hNfe2l1-luc*, respectively. As unexpected, ectopic *α*-Pal^NRF1^-V5 overexpression only caused a slight reduction in activity of *mNfe2l1-luc* reporter (Figure 5(a)), albeit this reporter gene was significantly induced by tBHQ (Figure 5(b), *b1*). Similarly, activity of *hNfe2l1-luc* reporter was substantially activated by tBHQ (Figure 5(b), *b2*) but significantly suppressed by ectopic expression of *α*-Pal^NRF1^-V5 (Figure 5(c), *c1* and *c3*). Such overexpression of ectopic *α*-Pal^NRF1^-V5 also led to similar transcriptional repression of *GSTa2*-*ARE×6-luc* reporter (Figure 5(c), *c2* and *c3*). These data indicate a possible contribution of *α*-Pal^NRF1^ to transrepression of *Nfe2l1*-*luc* and *ARE×6-luc* reporter genes.

Western blotting of *α*-Pal^NRF1^-expressing cells revealed that its targets TFAM and SOD1 were evidently enhanced, whilst abundances of Nfe2l1 and Nfe2l2 proteins were markedly diminished by ectopic *α*-Pal^NRF1^-V5 (Figure 5(d), *d1-d5*). Accordingly, HO-1 and GCLM abundances were also partially inhibited by *α*-Pal^NRF1^-V5 (Figure 5(d), *d6* and *d7*). Conversely, knockdown of *α*-Pal^NRF1^ by its specific siRNA (Figure 5(e) and M1) only caused a marginal reduction of TFAM (Figure 5(f) and M2), implying coordinated transcription regulation of human TFAM by *α*-Pal^NRF1^ with another factor (e.g., GABP^NRF2^). By contrast, both mRNA and protein levels of SOD1 were modestly promoted by silencing of *α*-Pal^NRF^ (Figure 5(g) and M3), implying at least one of other putative transcription factors competitively against knockdown of *α*-Pal^NRF1^. Similar results were also obtained from the case of *COX5a* (Figure 5(h)).

Further examinations revealed that modestly increased proteins of Nfe2l1, Nfe2l1, and HO-1 (Figure 5(m), *M4–6*) were coincidently accompanied by marginal enhancements of their mRNA expression levels, particularly following transfection of HepG2 cells with 50 nM siRNA against *α*-Pal^NRF1^ (Figures 5(i)–5(k)). In addition, GCLM protein and mRNA levels were almost unaffected by silencing of *α*-Pal^NRF1^ (Figure 5(l) and M7). Together, these results indicate that *α*-Pal^NRF1^ contributes to negative regulation of human Nfe2l1, Nfe2l2, and HO-1 (Figure 5(n)). However, no dose responses of Nfe2l1, Nfe2l2, and HO-1 to different concentrations of *α*-Pal^NRF1^-silenced siRNAs indicate a possible involvement of other transcription factors (e.g., Pitx2 and GABP^NRF2^). Moreover, *α*-Pal^NRF1^ also exerts disparate effects on GCLM, possibly depending on whether Nfe2l1, Nfe2l2, and other unidentified transcription factors were also stimulated by *α*-Pal^NRF1^ overexpression or its RNA-silencing.

### 2.9. Identification of Pitx2 as an Upstream Regulator to Mediate Transactivation of Human *Nfe2l1* Gene

**Figure 6:**
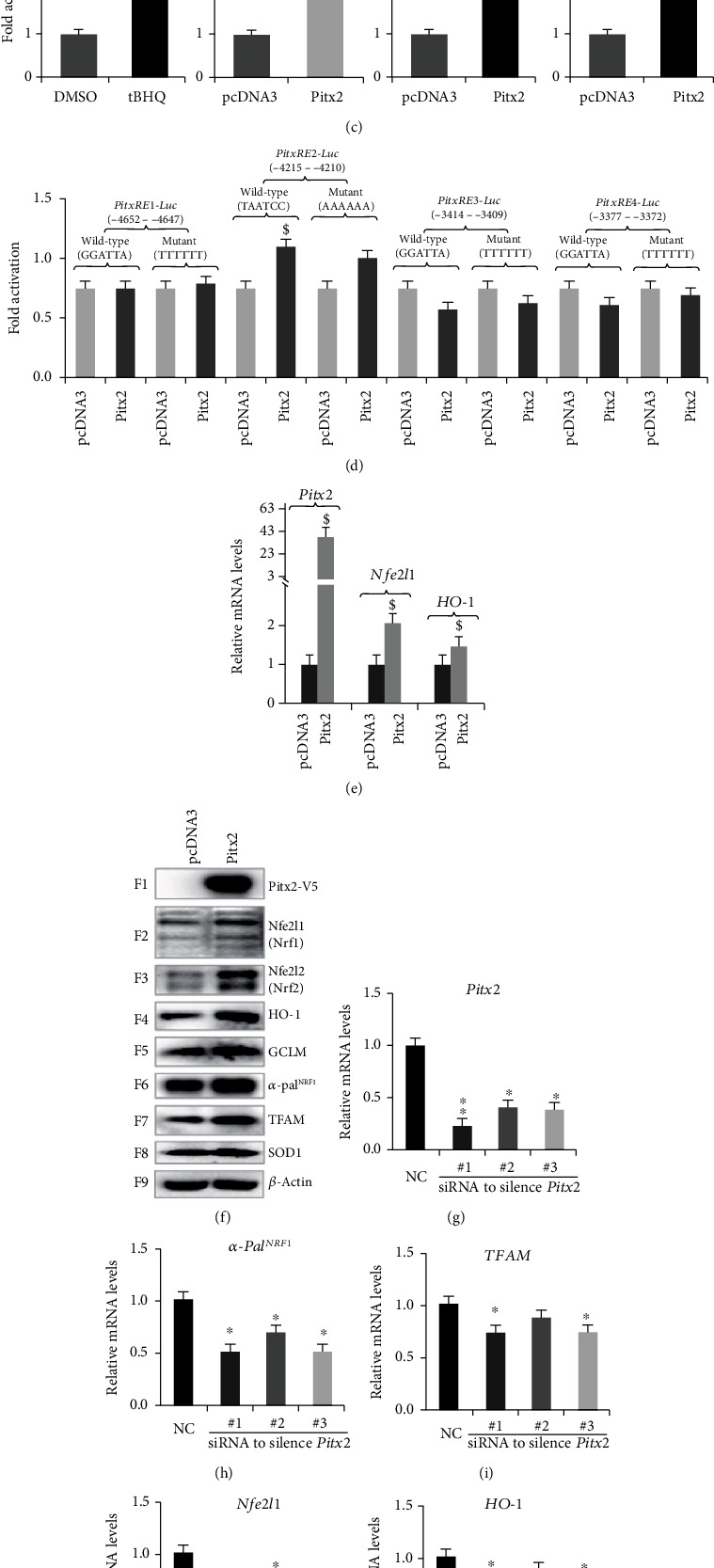
Identification of pitx2 as an upstream regulator of *Nfe2l1*, besides *α-pal^NRF1^*. (a) HepG2 cells were cotransfected for 8 h with *mNfe2l1-luc* (*A1*) or empty *pGL3-Basic* (*A2*), together with the pRL-TK control, plus a Pitx2 expression construct or an empty pcDNA3.1, and then allowed for a 24 h recovery before the luciferase activity was measured. The data are shown as mean ± SEM (*n* = 3 × 3) with significant increases ($, *p* < 0.01) or NS (not significance). (b) Putative *cis*-regulatory binding sites for Pitx2, *α*-Pal^NRF1^, and AREs within the human *Nfe2l1* promoter region were indicated. Various lengths of *hNfe2l1-luc* were cloned into the pGL3-Basic vector as shown schematically. (c) HepG2 cells were cotransfected for 8 h (i) with *hNfe2l1-luc* and pRL-TK and also treated for 24 h with 50 *μ*mol/L tBHQ or the DMSO vehicle (*C1*); (ii) with *hNfe2l1-luc* (*C2*), *hNfe2l1-luc1* (*C3*), or *hNfe2l1-luc2* (*C4*), along with the pRL-TK control, plus a Pitx2 expression construct or an empty pcDNA3.1, before being allowed for additional 24 h recovery. The samples were then subjected to dual luciferase assays. The results are shown as mean ± SEM (*n* = 3 × 3) with significant increases ($, *p* < 0.01). (d) HepG2 cells were cotransfected for 8 h with each of *PitxRE-luc* reporters or their mutants, together with pRL-TK plus a Pitx2 expression construct or an empty pcDNA3.1, and then allowed for 24 h recovery. Thereafter, the reporter activity was detected and calculated as mean ± SEM (*n* = 3 × 3) with significant increases ($, *p* < 0.01). (e) Distinct mRNA levels of *Pitx2*, *Nfe2l1*, and *HO-1* were detected by real-time qPCR of HepG2 cells that had been transfected with an expression construct for Pitx2 or an empty pcDNA3.1 vector. (f) Pitx2-expressing HepG2 cells were subjected to Western blotting of Pitx2, Nfe2l1, Nfe2l2, HO-1, GCLM, *α*-Pal^NRF1^, TFAM, and SOD1. (g–n) Distinct mRNA levels of *Pitx2* (g), *α-Pal^NRF1^* (h), *TFAM* (i), *Nfe2l1* (j), *HO-1* (k), *GCLM* (l), *COX5a* (m), and *SOD1* (n) were determined by real-time qPCR analysis of HepG2 cells that had been transfected with three different siRNAs against *Pitx2*. The results are shown as mean ± SEM (*n* = 3 × 3) with significant decreases (^∗^*p* < 0.01, ^∗∗^*p* < 0.001). (o) A model is proposed for effects of Pitx2 on Nfe2l1, Nfe2l2, *α*-Pal^NRF^, TFAM, and other genes in HepG2 cells.

Clearly, *α*-Pal^NRF1^ was identified as a direct target of Pitx2/3 (pituitary homeobox 2/3, also called paired like homeodomain 2/3) [38]. Here, we determine whether Pitx2 also acts as a direct upstream factor to mediate transactivation of human *Nfe2l1.* As shown in Figure 6(a), activity of *mNfe2l1-luc* reporter was significantly transactivated by the ectopic Pitx2 expression, when compared with the basal activity measured from its cotransfection with an empty pGL3-Basic vector. Similar results were also obtained from those *hNfe2l1-luc* reporters (Figures 6(b) and 6(c)). Of note, overexpression of ectopic Pitx2 caused gradual increments in the transactivity of *hNfe2l1-luc*, *hNfe2l1-luc1*, and *hNfe2l1-luc2* reporters (Figure 6(c), *C2-C4*). The *hNfe2l1-luc* activity was also strikingly induced by tBHQ to the extent of *hNfe2l1-luc1* transactivation mediated by Pitx2 (Figure 6(c), *cf. C1 with C3*). In addition, four distinct consensus Pitx-response elements (PitxREs), which are situated at the upstream of the indicated promoter region to have yielded *hNfe2l1-luc1* (Figure 6(b)), were also identified herein. The results revealed that only *PitxRE2-luc* activity was modestly activated by Pitx2, but the basal activity of *PitxRE3-luc* and *PitxRE4-luc* was partially suppressed by Pitx2 (Figure 6(d)). Together, these indicate that the transcriptional expression of human *Nfe2l1* gene is likely coregulated by Pitx2 and its target factors (e.g., *α*-Pal^NRF1^).

RT-qPCR analysis revealed that the endogenous mRNA expression of human *Nfe2l1* and *HO-1* was increased by Pitx2 overexpression (Figure 6(e)). Western blotting of Pitx2-expressing cells unraveled significant increases in abundances of all 7 examined proteins Nfe2l1, Nfe2l2, HO-1, GCLM, *α*-Pal^NRF1^, TFAM, and SOD1 (Figure 6(f), *F2-F8*). These demonstrate that Pitx2 may also serve as an upstream regulator to mediate transcriptional expression of Nfe2l1 (and Nfe2l2), besides *α*-Pal^NRF1^. This notion is further supported by the evidence obtained from silencing of Pitx2 by its three distinct siRNA sequences (as listed in Table S1). Consequently, silencing of Pitx2 led to varying decreases in basal mRNA expression levels of *α-Pal^NRF1^*, *TFAM*, *Nfe2l1*, *HO-1*, *GCLM*, *COX5a*, and *SOD1* to less extents than their equivalent controls (Figures 6(g)to 6(n)). Thereby, these results unveil that Pitx2 is required for mediating transcriptional expression of *Nfe2l1*, *α-Pal^NRF1^*, and their target genes (Figure 6(o)).

### 2.10. Identification of TFAM as another Direct Cotarget of Human Nfe2l1, Nfe2l2, and Pitx2

**Figure 7:**
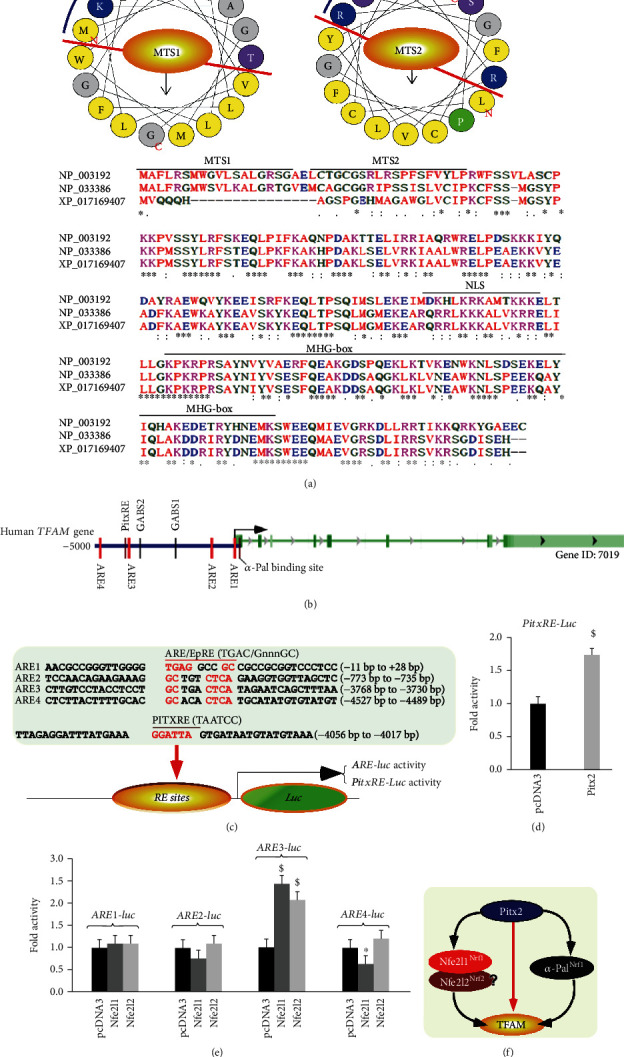
Transcriptional regulation of *TFAM* by Nfe2l1, Nfe2l2, and Pitx2. (a) Two similar *α*-helical structural wheels were formed by successive mitochondria-targeting sequences MTS1 (aa 1-18) and MTS2 (aa 21-38). Basic arginine and lysine residues were placed on blue backgrounds; nucleophilic serine and threonine residues are on purple backgrounds; an unamiable proline residue was on a green background, and all other hydrophobic amino acids were on yellow backgrounds, except for small alanine and glycine on grey backgrounds. The *lower panel* shows an alignment of three amino-acid sequences of human TFAM (NP_003192) and mouse TFAMs (NP_033386 and XP_017169407), in which, MTS1, MTS2, nuclear localization signal (NLS), and DNA-binding MHG-box were indicated. (b) The putative consensus ARE sites and other *cis*-regulatory binding sites for Pitx2, *α*-Pal^NRF1^, or GABP within the human *TFAM* gene promoter region were indicated. (c) Four distinct *ARE*-driven (i.e., *ARE1*-*luc* to *ARE4-luc*) and another *Pitx2RE-luc* reporters were constructed into the pGL3-Promoter vector. (d) HepG2 cells were cotransfected with *PitxRE-luc* and pRL-TK, plus a Pitx2 expression plasmid or an empty pcDNA3.1, and then allowed for 24 h recovery before the luciferase activity was measured. The data are shown as mean ± SEM (*n* = 3 × 3) with significant increase ($, *p* < 0.01). (e) HepG2 cells were cotransfected with each of *ARE1*-*luc* to *ARE4-luc*, together with pRL-TK plus an expression construct for Nfe2l1, Nfe2l2, or an empty pcDNA3.1, and then allowed for 24 h recovery, before such *ARE*-driven activity was detected. The resultant data are shown as mean ± SEM (*n* = 3 × 3) with significant increases ($, *p* < 0.01) or decreases (^∗^*p* < 0.01). (f) A model is proposed to explain the transcriptional regulation of TFAM by Nfe2l1, Nfe2l2, Pitx2, and *α*-Pal^NRF1^ in HepG2 cells.

Although TFAM is known as a direct target of *α*-Pal^NRF1^ [18, 45, 46], we further determined whether it also serves as another direct cotarget of Nfe2l1, Nfe2l2, and/or Pitx2. As illustrated in Figure 7(a) (*upper panel*), two similar *α*-helical structures were wheeled, respectively, by the successive mitochondria-targeting sequences (MTS1 and MTS2) in each nuclearly encoded TFAM. Alignment of three amino acid sequences from human and mouse showed that MTS1 (aa 1-18) and MTS2 (aa 21-38) are located within the N-terminal region of this MHG- (high mobility group-) box-binding transcription factor TFAM (Figure 7(a), *lower panel*). Further bioinformatic analysis revealed several putative consensus *cis*-regulatory elements, such as *AREs*, *PitxRE*, and *α-Pal*-binding site within the promoter region of human *TFAM* (Figure 7(b)). Amongst them, *ARE*- and *PitxRE*-driven luciferase reporters were established herein (Figure 7(c)). As a result, the luciferase assays of both *PitxRE-Luc* activity (Figure 7(d)) and *ARE3-Luc* activity (Figure 7(e)) demonstrated that the transcriptional expression of TFAM is, *de facto*, coregulated by human Pitx2, Nfe2l1, and Nfe2l2 (Figures 7(d)–7(f)). Together with the previous work by L'Honore et al. [38], these demonstrate that *TFAM* is a *bona fide* cotarget gene of Nfe2l1, Nfe2l2, and *α*-Pal^NRF1^, as well as their upstream regulator Pitx2 (Figure 7(f)).

### 2.11. Distinct Contributions of Human Nfe2l1 and Nfe2l2 to Differential Expression of TFAM, PGC-1*α*/*β*, and Associated Genes That Are Involved in the Nuclear-to-Mitochondrial Communication

**Figure 8:**
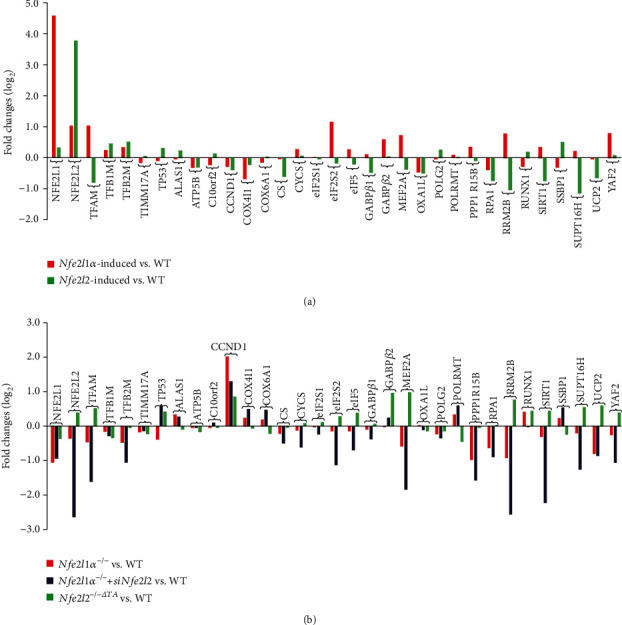
Distinct or even opposite contributions of Nfe2l1 and Nfe2l2 to expression of TFAM, TFB1M, TFB2M, and other critical genes for the nuclear-to-mitochondrial communication. (a) 33 of differential expression genes (DEGs) were selected from RNA-sequencing of either *Nfe2l1α*-induced and Nfe2l2-induced cell lines versus their WT controls (by the value of FDR < 0.05). (b) Those gene expression levels were also determined by RNA-sequencing of *Nfe2l1α^−/−^*, *Nfe2l1α^−/−^+siNfe2l2*, and *Nfe2l2^-/-ΔTA^* cell lines. Their values were then calculated by Log2 (fold change), relative to their equivalents of wild-type (WT) control cells.

Since TFAM is of crucial importance in the nuclear control of the mitochondrial function, it is also postulated to be involved in redox regulation by Nfe2l1 and Nfe2l2. As anticipated, Figure 8(a) showed that *TFAM* was upregulated by Nfe2l1, but downregulated by Nfe2l2, in their respective tetracycline-inducible cell systems (as described by Wang et al. [47]), albeit both CNC-bZIP factors positively contributed to upregulation of *TFB1M* and *TFB2M*. These were corroborated by further evidence obtained from *Nfe2l1α^−/−^*, *Nfe2l1α^−/−^+siNfe2l2*, and *Nfe2l2^-/-ΔTA^* cell lines (Figure 8(b)).

It was found that Nfe2l1 and Nfe2l2 yielded similar contributions to downregulation of *ATP5B* (ATP synthase F1 subunit *β*), *COX4I1* (cytochrome c oxidase subunit 4I1), *OXA1L* (oxidase cytochrome c assembly 1-like), *CCND1*, or *RPA1* (Figure 8(a)). However, Nfe2l1 and Nfe2l2 also led to differential or opposite expression of other 20 genes, including ALAS1 (5′-aminolevulinate synthase 1), *C10orf2* (twinkle mtDNA helicase), *CS* (citrate synthase), *CYCS* (cytochrome c, somatic), *POLG2* (DNA polymerase *γ*2), *RRM2B* (ribonucleotide reductase subunit M2B), *SSBP1* (single stranded DNA binding protein 1), *eIF2S1* (*eIF2α*), *eIF2S2* (*eIF2β*), *eIF5*, *GABPβ1*, *GABPβ2*, *MEF2A* (myocyte enhancer factor 2A), *PPP1R15B*, *RUNX1*, *SIRT1*, *SUPT16H*, *UCP2*, and *YAF2* (YY1-associated factor 2). Reversely, changes in these gene expression levels were also determined by RNA-sequencing of *Nfe2l1α^−/−^*, *Nfe2l1α^−/−^+siNfe2l2*, and *Nfe2l2^-/-ΔTA^* cell lines (Figure 8(b)).

**Figure 9:**
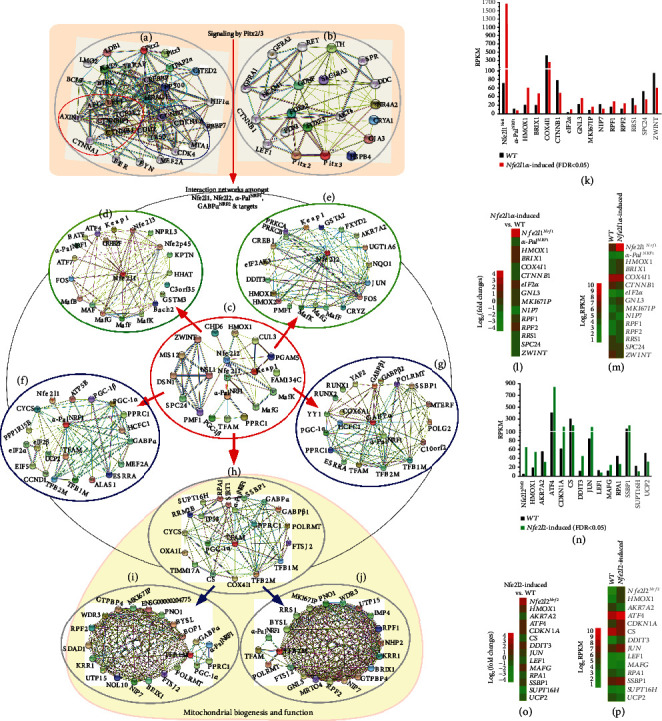
A hierarchical interaction network integrated with those relevant genes involved in the nuclear-to-mitochondrial communication. (a, b) Two interaction subnetworks with Pitx2- and Pitx3-regulated genes. (c–e) A core subnetwork of redox-relevant genes regulated by Nfe2l1 and/or Nfe2l2, together with Nfe2l1- and/or Nfe2l2-interactors in additional two extended subnetworks. (f–j) Five subnetworks are monitored by *α*-Pal^NRF1^, GABP*α*^NRF2^, TFAM, TFB1M, and/or TFB2M, which are key players as the nuclear controls of mitochondrial biogenesis and function. (k–m) Differentially expressed genes (DEGs) are contributed by the Nfe2l1*α*-inducible expression. Their FPKM values (FDR < 0.05) were obtained from RNA-sequencing of Nfe2l1*α*-induced cells versus WT cells. Such DEGs were also shown by two distinct heat maps, as scaled in different ways. (n–p) 14 of DEGs are regulated by Nfe2l2-inducible expression, which are also shown in two distinct fashions as described above.

An overview of multilayer interaction networks with the aforementioned key molecular nodes is illustrated in Figures 9(a)–9(j), to provide an explicit understanding of the nuclear-to-mitochondrial communication by multiple extranuclear signaling directed towards the nuclear controls of the mitochondrial respiration, energy metabolism, and redox regulation. Such hierarchical network consists of several subnetworks, with each of important transcription factors serving a hub in the center. Of note, the mitochondrial genome is transcriptionally controlled by TFAM, TFB1M, and TFB2M (Figures 9(h) and 9(j)), although they are nucleus-encoded, and also controlled by *α*-Pal^NRF1^, GABP*α*^NRF2^, Nfe2l1^Nrf1^, and/or Nfe2l1^Nrf2^ (c–g). Those differentially expressed genes (DEGs) as key players in this network were further analyzed by RNA-sequencing of *Nfe2l1α*-induced cells (Figures 9(k)to9(m)) and *Nfe2l2*-induced cells (n–p), when compared with their *WT* controls.

The result validated that HO-1 (encoded by HMOX1) is a direct cotarget of Nfe2l1 and Nfe2l1, because similar increased mRNA expression levels were attributed to induction of either CNC-bZIP factor (Figures 9(k)–9(p)). Particularly, 13 of DEGs were bidirectionally regulated by Nfe2l1 (Figures 9(k)–9(m)), 6 of which *BRIX1* (for biogenesis of ribosomes), *eIF2α*, *GNL3* (G protein nucleolar 3), *MKI67IP* (a nucleolar protein interacting with the FHA domain of MKI67), *RPF1* (ribosome production factor 1), and *RPF2* were promoted by Nfe2l1, but as accompanied by downexpression of 7 genes *α-Pal^NRF1^*, *COX4I1*, *CTNNB1* (catenin-*β*1), *NIP7* (a nucleolar pre-rRNA processing protein), *RRS1* (ribosome biogenesis regulator 1), *SPC24* (a component of NDC80 kinetochore complex), and *ZWINT* (ZW10 interacting kinetochore protein). Further analysis revealed that Nfe2l2 contributed to 12 of DEGs, besides HMOX1 (Figures 9(n)–9(p)). Amongst them, *ATF4*, *CDKN1A* (p21), *DDIT3* (Chop), *Jun*, *MafG*, and *SSBP1* were activated by Nfe2l2, while *AKR7A2* (aldo-keto reductase family 7 member A2), *CS*, *LEF1*, *RPA1*, *SUPT16H*, and *UCP2* (uncoupling protein 2) were depressed by this CNC-bZIP factor.

**Figure 10:**
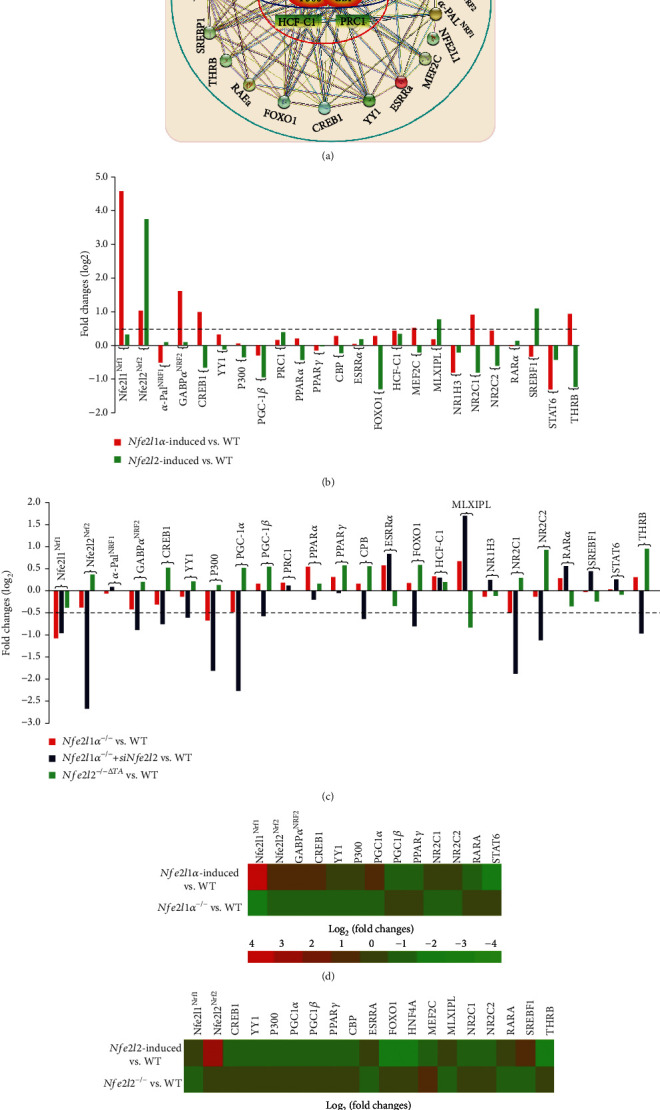
Nfe2l1 and Nfe2l2 contribute to differential expression of PGC-1*α*/*β* and associated genes. (a) The PGC-1*α*/*β*-centered interaction network with distinct families of 21 transcription factors and additional 4 cofactors, which was constructed by the STRING tool (https://string-db.org/). (b) The Log2 (fold change) values of those indicated genes were calculated by comparison of RNA-sequencing data obtained from either *Nfe2l1α*-induced or *Nfe2l2*-induced cells with equivalent wild-type (WT) controls. (c) Similar Log2 (fold change) values of the above-described genes were also calculated by comparison of RNA-sequencing data obtained from *Nfe2l1α^−/−^*, *Nfe2l1α^−/−^+siNfe2l2*, or *Nfe2l2^-/-ΔTA^* cell lines versus their equivalent *WT* controls (FDR < 0.05). (d) A heat map shows differential expression levels of Nfe2l1*α*-affected genes determined by comparison of either *Nfe2l1α^−/−^* or *Nfe2l1α*-induced cells with the respective *WT* controls (FDR < 0.05). Such different colored genes were scaled by their Log2 values. (e) Another heat map of 19 Nfe2l2-affected genes, which were determined by comparison of *Nfe2l2^-/-ΔTA^* or *Nfe2l2*-induced cells with their respective *WT* controls (FDR < 0.05).

Since PGC-1*α*/*β* and PRC1 are accepted as key transcription cofactors involved in the nuclear control of mitochondrial biogenesis and function, here we established an interaction network, with distinct families of 21 transcriptional factors and 6 cofactors, by using the STRING tool (Figure 10(a)). Differential expression levels of these genes possibly affected by Nfe2l1 or Nfe2l2 were determined by comparison of the RNA-sequencing data of either *Nfe2l1α-* or *Nfe2l2-*induced cells versus *WT* controls (Figure 10(b)) and also of *Nfe2l1α^−/−^*, *Nfe2l1α^−/−^+siNfe2l2*, or *Nfe2l2^-/-ΔTA^* cell lines versus another *WT* controls (Figure 10(c)). Further comparison of their data from *Nfe2l1α*-induced and *Nfe2l1α^−/−^* cell lines revealed that the opposite expression of 13 relevant genes was dictated by a difference between the presence and absence of this CNC-bZIP factor (Figure 10(d)). There exists a positive relationship of Nfe2l1*α* with *GABPα^NRF2^*, *CREB1*, *YY1*, *P300*, *PGC-1α*, *NR2C1* (nuclear receptor subfamily 2 C1, with its ligand binding domain belonging typically to the steroid/thyroid hormone receptor superfamily) and *NR2C2*, but as companied by its another negative relationship with *PGC-1β*, *PPARγ*, *RARA* (retinoic acid receptor *α*), and *STAT6* (Figure 10(d)). In the same network, 19 opposite expression genes affected by Nfe2l2 were selected by comparative analysis of *Nfe2l2-*induced and *Nfe2l2^-/-ΔTA^* cell lines (Figure 10(e)). Among them, a positive effect of Nfe2l2 on *Nfe2l1*, *ESRRA* (estrogen related receptor *α*), *MLXIPL* (MLX interacting protein like), *RARA*, and *SREBF1* (sterol regulatory element binding protein 1) was also accompanied by its another negative relationship with *CREB1*, *CREBBP* (CREB-binding protein, called CBP), *YY1*, *P300*, *PGC-1β*, *PPARα*, *PPARγ*, *FOXO1*, *HNF4A*, *MEF2C*, *NR2C1*, *NR2C2*, and *THRB* (thyroid hormone receptor *β*). Together, these indicate that *PGC-1α* and *STAT6* are Nfe2l1*α*-specific, while 9 of Nfe2l2-specific genes are *ESRRA*, *MLXIPL*, *SREBF1*, *PPARA*, *CBP*, *FOXO1*, *HNF4A*, *MEF2C*, and *THRB.* Rather, Nfe2l1*α* and Nfe2l2 have an overlapping effect to downregulate *PGC-1β* and *PPARγ*, apart from their opposite effects of both CNC-bZIP factors to bi-directionally regulate *CREB1, YY1, EP300, NR2C1, NR2C2* and *RARA*.

## 3. Discussion

**Figure 11:**
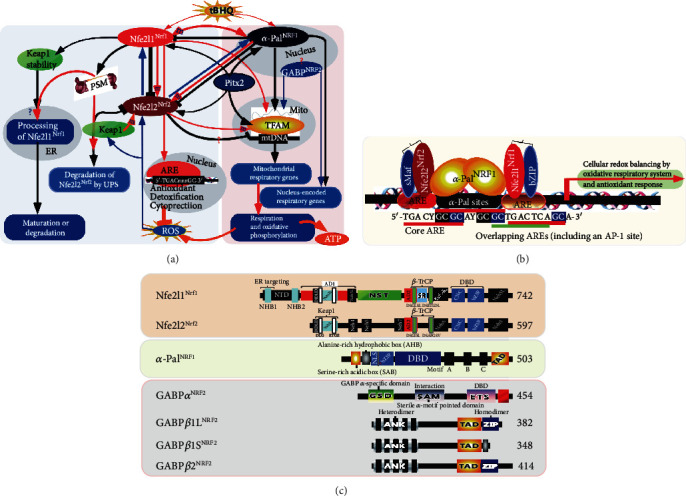
An integral model to interpret coordinated regulation of distinct cellular respiratory and antioxidant gene transcription networks. (a) A model is based on our experimental evidence, to give a better understanding of regulatory cross-talk among Nfe2l1^Nrf1^, Nfe2l2^Nrf2^, *α*-Pal^NRF1^, and Pitx2, all targeting TFAM. During the nuclear-to-mitochondrial communication, Nfe2l1^Nrf1^ makes opposing contributions to bidirectional regulation of Nfe2l2^Nrf2^ and *α*-Pal^NRF1^ by itself and target proteasome (*PSM*) at two distinct layers. Nfe2l2^Nrf2^ can determine posttranscriptional regulation of Nfe2l1^Nrf1^ and *α*-Pal^NRF1^, but the detailed mechanism remains unclear. In turn, mouse *α*-Pal^NRF1^ contributes to positive regulation of Nfe2l1^Nrf1^ and Nfe2l2^Nrf2^, although no canonic GC-rich *α*-Pal-binding sites exist in these mouse CNC-bZIP gene promoter regions (Table S2). Contrarily, human *α*-Pal^NRF1^ makes a negative contribution to transcriptional expression of Nfe2l1^Nrf1^ and Nfe2l2^Nrf2^ (the latter Nfe2l2^Nrf2^ is dominantly negatively regulated by human Nfe2l1^Nrf1^), albeit all three factors are activated by redox inducer tBHQ. In addition to negative regulation of Nfe2l2^Nrf2^ by Keap1, the adaptor subunit of Cullin 3-based E3 ubiquitin ligase can also make a positive contribution to transcriptional regulation of mouse Nfe2l2^Nrf2^, rather than Nfe2l1^Nrf1^, as found in MEFs. However, human Nfe2l1^Nrf1^ is essential for stabilization of Keap1, but it is unknown whether this adaptor protein is involved in the proteolytic processing of Nfe2l1^Nrf1^. Notably, the nucleus-controlled mitochondrial respiratory and oxidative phosphorylation are also a primary source of ROS in cells, which triggers activation of Nfe2l1^Nrf1^, Nfe2l2^Nrf2^, and *α*-Pal^NRF1^ to certain extents so that cellular redox homeostasis is maintained at a steady state. Besides, GABP^NRF2^ is also required for this process, but possible cross-talks of this ETS family factor with Nfe2l1^Nrf1^ and Nfe2l2^Nrf2^ are not yet identified here. The “M”-marked arrowheads indicate those activity in the mouse but not in the human; such distinction was also shown (in Figure S2). (b) Schematic explanation of the intracellular redox homeostasis balanced by an oxidative respiratory system and another antioxidant cytoprotective response. Most of *ARE*-driven genes are transcriptionally regulated by distinct functional heterodimers of either Nfe2l1^Nrf1^or Nfe2l2^Nrf2^ with sMaf or other bZIP proteins, whilst most of the nucleus-encoded mitochondrial respiratory genes are controlled predominantly by *α*-Pal^NRF1^ homodimers. Of note, the GC-enriched *α*-Pal-binding site is overlapped with the -GC-motif of *ARE-core* sequences (each of which contains an AP-1 site). This implies synergistic and/or antagonistic regulatory effects of Nfe2l1^Nrf1^, Nfe2l2^Nrf2^, and *α*-Pal^NRF1^ on certain expression of distinct cognate target genes. (c) Schematic representation of distinct structural domains of Nfe2l1^Nrf1^, Nfe2l2^Nrf2^, and *α*-Pal^NRF1^, as well as GABP*α*^NRF2^, GABP*β*1L^NRF2^, GABP*β*1S^NRF2^, and GABP*β*2^NRF2^. Of note, all domains and motifs of Nfe2l1^Nrf1^ and Nfe2l2^Nrf2^ were defined (37), but neither have no homology with *α*-Pal^NRF1^, GABP*α*^NRF2^, and GABP*β*^NRF2^. Distinct domains and motifs of these nuclear respiratory factors are identified by bioinformatic analysis of their amino-acid sequences. ANK: ankyrin repeats; DBD: DNA-binding domain; ER: endoplasmic reticulum; ETS: E26 transformation specific; GSD: GABP*α*-specific domain; NLS: nuclear localization signal; Mito: mitochondria; SAM: sterile *α*-motif pointed domain; TAD: transactivation domain.

In the present study, we have, for the first time, provided a better interpretation of two totally distinctive Nrf1 transcription factors (i.e., Nfe2l1^Nrf1^ and *α*-Pal^NRF1^, Figures 11(a) and 11(b)). This is an attempt to rectify the previously confusing explanations of both Nrf1 factors, from their commonly shared abbreviations in the literature as far as we know, in which one of the most typical representative is the publication confused by L'Honore et al. [38]. Similarly, another two similar Nrf2 transcription factors [48, 49] are also referred to as Nfe2l2^Nrf2^ and GABP*α*^NRF2^, respectively. Of note, the antioxidant master Nfe2l2^Nrf2^ is highly conserved with Nfe2l1^Nrf1^, but neither have evolutionary conservation with those nuclear respiratory factors *α*-Pal^NRF1^ and GABP*α*^NRF2^, of which the latter two factors have also no any homology with each other (Figure 11(c)). Collectively, our present study has clearly elucidated both synergistic and antagonistic roles of Nfe2l1^Nrf1^, Nfe2l2^Nrf2^, and *α*-Pal^NRF1^ in integrative regulation of multiple signaling to the nuclear-to-mitochondrial respiratory and antioxidant transcription networks, which differ between the mouse and human, though (also see Figure S2).

### 3.1. Synergism of Mouse Nfe2l1^Nrf1^, Nfe2l2^Nrf2^, and *α*-Pal^NRF1^ to Coordinate the Nuclear-to-Mitochondrial Respiratory and Antioxidant Gene Responses in MEFs

Clearly, Nfe2l1 and Nfe2l2 are two highly conserved members of the CNC-bZIP transcription factors, which predominantly regulate distinct subsets of *ARE*-driven cytoprotective genes against cellular oxidative stress [37, 39]. These target genes are critically involved in maintaining cellular homeostasis and organ integrity during normal development and growth, as well as healthy life process, in which Nfe2l1 and Nfe2l2 cannot only exert their combinational and/or overlapping roles, but also manifest their distinctive functions from each other. Importantly, Nfe2l1 elicits its unique indispensable physio-pathological functions, because its loss of function in the mouse leads to distinct pathological phenotypes, even in the presence of Nfe2l2 [37]. This implies a fact that the loss of Nfe2l1 cannot be compensated by Nfe2l2, albeit Nfe2l2 has been accepted as a master regulator of antioxidant, detoxification, and cytoprotective responses [39, 50]. Such significant distinctions in the functioning of between Nfe2l1 and Nfe2l2 are dictated by their respective intrinsic features.

Interestingly, Nfe2l1, but not Nrf2, with several domains being more highly conserved with its ancestral CNC, Skn-1, and Nach proteins [51], is located in the ER and its connected nuclear envelope membranes. Of note, there exist two extra functional domains (i.e., NTD and NST) within Nfe2l1, rather than Nfe2l2. The ER-targeting NTD of Nfe2l1 enables it to be topologically anchored within and around the membranes [52, 53], whilst its NST glycodomain facilitates its proper protein folding and subsequent processing [42, 54]. The membrane-topology of Nfe2l1 determines the vectorial selective proteolytic processing of this CNC-bZIP protein by proteasomes and/or other cytosolic proteases in close proximity to the ER, in order to yield distinct lengths of its isoforms, one of which is mature CNC-bZIP factor translocated into the nucleus [42, 53]. Such the membrane-bound Nfe2l1 factor can also be activated in the unfolded protein response to ER stress stimulated by tunicamycin (to inhibit its N-linked glycosylation) [55], in addition to its proteasomal “bounce-back” response [42, 56]. By sharp contrast, the water-soluble Nfe2l2 is segregated by Keap1 within the cytoplamic compartments, where it is targeted for its ubiquitination-mediated proteasomal degradation [39]. Rather, upon stimulation of Nfe2l2 by oxidative stress, this CNC-bZIP factor is dissociated from the cytoplamic Keap1, so that it is subsequently translocated into the nucleus and transactivates ARE-driven target genes. Collectively, these distinctions of Nfe2l1 from Nfe2l2 demonstrate disparate selection of their tempospatial activation or inactivation insomuch as to finely tune distinct subsets of target genes. As such, this also presages synergism and antagonism of between Nfe2l1 and Nfe2l2 in regulating distinct cognate gene expression.

In fact, mouse *Nfe2l1^−/−^* embryonic lethality occurred at middle to late gestation starting at E13.5 [57], whereas double knockout of *Nfe2l1^−/−^*:*Nfe2l2^−/−^* caused an earlier death of the resulting mouse embryos at E10.5 due to extensively increased apoptosis and growth retardation induced by severe endogenous oxidative stress [58]. The elevated ROS levels were resulted from severely impaired expression of antioxidant defense genes (e.g., *Mt-1*, *Gclm*, *Gclc*, *Ferritin H*, *Ho-1*, and *Nqo1*) in *Nfe2l1^−/−^:Nfe2l2^−/−^* MEFs, when compared with their individual knockout of *Nfe2l1^−/−^* or *Nfe2l2^−/−^*. Hence, it is inferred that Nrf2 is also allowed for a partial compensation for the loss of Nrf1's function in synergistically regulating critical genes for the intracellular redox homeostatic setting at the robust threshold state during embryogenesis. Such partially overlapping functions of both Nfe2l1 and Nfe2l2 are determined by coexpression patterns of the two CNC-bZIP factors [59–61] and their similarity of conserved sequences [37], which are also, though, driven by their respective *ARE*-containing gene promoters (Table S2).

It is, to our surprise, that the evidence presented herein reveals that knockout of *Nfe2l1* in mice leads to significant decreases of both mRNA and protein levels of Nfe2l2 expressed in *Nfe2l1^−/−^* MEFs. By contrast, almost no changes in mRNA expression levels of *Nfe2l1* in *Nfe2l2^−/−^* MEFs, but the loss of *Nfe2l2* results in obvious decreases in abundances of the full-length Nfe2l1*α* and its processed proteins (i.e., isoform-B, C, and D), although this was accompanied by an increased Nfe2l1*β*. Collectively, these results demonstrate that Nfe2l1 acts as a dominant positive regulator to monitor the basal constitutive expression of Nfe2l2, at least in MEFs (Figure S2A). In turn, the latter Nfe2l2 does not control transcription of mouse *Nfe2l1* gene, but it is required for stabilization of Nfe2l1*α* and its proteolytic processing to yield a mature CNC-bZIP factor. This notion is further substantiated by the evidence obtained from *Keap1^−/−^* MEFs, showing that accumulation of Nfe2l2 is accompanied by significant increases in abundances of Nfe2l1*α* and its derivates, but basal mRNA expression of *Nfe2l1* remains to be unaffected, whilst Nfe2l2 mRNA levels were decreased significantly.

Recently, emerging evidence showed that the regulatory cross-talks of Nfe2l2 (but Nfe2l1 was not shown) with either *α*-Pal^NRF1^ or GABP*α*^NRF2^ are involved in the nuclear control of mitochondrial biogenesis and relevant biological functions [48, 49, 62]. In this study, we have unravelled that knockout of *Nfe2l1* causes significant decreases in mRNA and/or protein levels of *α*-Pal^NRF1^ and its target genes *TFAM*, *Ndufv1*, *Ndub6*, *COX5a*, and *SOD1* in *Nfe2l1^−/−^* MEFs, whereas knockout of *Nfe2l2^−/−^* also leads to marked decreases in protein abundances of *α*-Pal^NRF1^ and TFAM, but almost unaltered mRNA levels of both *α-Pal^NRF1^*and *TFAM* were observed in *Nfe2l2^−/−^* MEFs. These results demonstrate that Nfe2l1 acts as a dominant regulator to monitor transcriptional expression of *α*-Pal^NRF1^, TFAM, and other target genes in MEFs, whilst stability of both *α*-Pal^NRF1^ and TFAM proteins may also be monitored by a Nfe2l2-mediated mechanism. This notion is further supported by another evidence showing that *Keap1^−/−^* MEFs gave rise to evident increases in basal abundances of Nfe2l2, Nfe2l1*α*, and its processed isoforms, which were accompanied by obvious increased proteins of both *α*-Pal^NRF1^ and TFAM. However, only modest increases in their mRNA expression levels of *α*-Pal^NRF1^ and TFAM were examined in *Keap1^−/−^* MEFs, with an exception of significantly reduced mRNA levels of *Nfe2l2*, but not of *Nfe2l1*. This exception implies that the transcriptional expression of Nfe2l2 requires for another transcription factor involving a putative positive feedback mechanism regulated by Keap1, albeit this Nfe2l2 inhibitor negatively regulates stability of this CNC-bZIP protein. Moreover, our further luciferase assays also revealed that both Nfe2l1 and Nfe2l2 contribute to transcriptional expression of distinct ARE-driven *α-Pal^NRF1^* reporters. Conversely, marked decreases in mRNA and protein levels of Nfe2l1 and Nfe2l2 resulted from heterogeneous deletion of mouse *α-Pal^NRF1+/-^*. Overall, these indicate bidirectional cross-talks of Nfe2l1 and/or Nfe2l2 with *α*-Pal^NRF1^ at distinct layers to coordinate the extranuclear signaling directed towards the nuclear-to-mitochondrial respiratory and antioxidant transcription networks.

### 3.2. Cross-Talks between Human Nfe2l1^Nrf1^, Nfe2l2^Nrf2^, and *α*-Pal^NRF1^ to Coordinate the Nuclear-to-Mitochondrial Respiratory and Antioxidant Gene Transcription Networks

Intriguingly, we found almost no effects of *hNfe2l1^−/−^* or *hNfe2l2^−/−^* on mRNA and protein levels of human *α*-Pal^NRF1^ in this experimental setting, albeit both CNC-ZIP genes contain at least one consensus *α*-Pal binding site within their promoters (Table S3). This is also further confirmed by transcriptomics sequencing of *hNfe2l1^−/−^+siNfe2l2* cells, as compared with those obtained from either *hNfe2l1^−/−^* or *hNfe2l2^−/−^* cells. Such results are really contrary to those obtained from mouse *Nfe2l1^−/−^* and *Nfe2l2^−/−^* as described above. Conversely, significantly decreases of human Nfe2l1 and Nfe2l2 protein levels were caused by forced expression of ectopic *α*-Pal^NRF1^. Similarly, transcriptional activity of *hNfe2l1-luc*, rather than *mNfe2l1-luc*, reporters was also markedly diminished by ectopic *α*-Pal^NRF1^. By contrast, a recovery of human Nfe2l1 and Nfe2l2 from inhibition of *α*-Pal^NRF1^ was also acquired after this respiratory factor was silenced. Notably, silencing of *α*-Pal^NRF1^ can only cause a modest decrease in its target TFAM expression, implying that this mitochondrial transcription factor is also monitored by other factors (e.g., GABP*α*^NRF2^ and/or Pitx2) beyond *α*-Pal^NRF1^. In this study, we also found that TFAM was downregulated in *hNfe2l1^−/−^* cells (albeit with accumulation of Nfe2l2) and hence upregulated in *hNfe2l2^−/−^* cells. Our further evidence reveals that TFAM along with *α*-Pal^NRF1^ is significantly induced by the redox inducer tBHQ, but such inducible expression levels of both factors are almost completely abolished by *hNfe2l1^−/−^*. Collectively, these indicate that Nfe2l1 and Nfe2l2 contribute, respectively, to the putative positive and negative regulation of TFAM, even though both CNC-ZIP factors are inhibited by *α*-Pal^NRF1^ through an as-yet-unidentified mechanism (Figures 11(a) and S2B). Consistently, the positive regulation of TFAM by Nfe2l1 is further corroborated by its consensus ARE3-driven report assays. However, it should also be noted that the opposing effects of Nfe2l2 on the endogenous TFAM and its consensus *ARE3-luc* reporter could depend on distinct ARE3-adjoining promoter contexts of the genome backgrounds.

Importantly, we have presented the experimental evidence that activity of *hNfe2l1-luc* reporter is transactivated by Pitx2 (albeit it serves as a direct upstream regulator of *α*-Pal^NRF1^ [38]). Further evidence also reveals that endogenous expression of Nfe2l1 (and Nfe2l2) was significantly upregulated by overexpression of Pitx2, but rather downregulated by silencing of this homeobox factor. Such Pitx2-directed alternations of Nfe2l1 and Nfe2l2 are accompanied by corresponding changes of HO-1 and GCLM, as well as *α*-Pal^NRF1^, TFAM, and COX5a. In addition to human Nfe2l1, TFAM is another potential target of Pitx2, because its consensus *PitxRE-luc* reporter was transactivated by this homeobox factor. These, together with our previously reported evidence [44, 55], demonstrate multiple regulatory across-talks between Nfe2l1, Nfe2l2, *α*-Pal^NRF1^ and Pitx2, and their cotarget TFAM, are integrated by multiple extranuclear (e.g., ER-driven) signaling to the nuclear-to-mitochondrial controls of distinct cellular respiratory and antioxidant transcription networks, albeit they differ between the mouse and human (Figure S2B).

However, the nucleus-controlled mitochondrial respiratory and oxidative phosphorylation are also a primary source of endogenous ROS byproducts in eukaryotic cells. This facilitates to trigger physiological activation of Nfe2l1, Nfe2l2, and *α*-Pal^NRF1^ to certain extents in so much as that the intracellular redox homeostasis is maintained at a robust steady-state. Altogether, interaction between these transcription factors comprises a complex feedback circuit with hierarchical regulatory networks served to maintain robust redox homeostasis by balancing a distinct cellular oxidative respiratory system and an antioxidant cytoprotective response.

## 4. Conclusion

Generally, most biological functions and physio(patho)logical responses at different levels are determined by a range of regulatory mechanisms from various signaling pathways toward multiple transcription factor-mediated gene networks. Such being the case, these transcription factors and other regulatory molecules could be simplified as key modules which cross-talk amongst them, in order to form a hierarchical network. Nfe2l1 and Nfe2l2 are embedded in such a complex molecular interaction network, responsible for antioxidant, detoxification, and cytoprotective adaptation to distinct physio-pathological stresses during life process. Of note, Nfe2l1 is endowed with unique indispensable biological functions. This fact confers Nfe2l1 to be distinguished from Nfe2l2 at regulating distinct subsets of ARE-driven cognate genes (e.g., *Aldh1a1* and *GSTa1*). But, the inter-regulatory synergism of Nfe2l1 and Nfe2l2 confers both factors to exert their overlapping roles for cotarget genes (e.g., *MT-1*, *GSTp*, and *SOD1*) in MEFs. By sharp contrast, apparent synergistic and antagonistic relationships of between human Nfe2l1 and Nfe2l2 are also demonstrated. Nfe2l1 is a dominant repressor, whilst Nfe2l2 is thus negatively regulated by Nfe2l1 in HepG2 cells. Such function of Nfe2l1 is considered as a brake to avoid Nfe2l2 overshoot. For this simplification to be valid, there should always be a correlation between Nfe2l1 and Nfe2l2 so that both CNC-bZIP factors along with cognate target genes are finely tuned in order to meet the changing needs of cells under all conditions. In fact, Nfe2l1 and Nfe2l2 cannot be reduced as a simple module factor alone (Figure 9). Now, the question becomes what is the minimal modular network to reproduce the observed data and why it is so evolved. At least three of them *α*-Pal^NRF1^, TFAM, and Pitx2 should also be included, in addition to both Nfe2l1 and Nfe2l2. Overall, this study also redefines that the nuclear controls of mitochondrial respiration and biogenesis, as well as certain protein synthesis and degradation, are physiologically integrated with multiple extranuclear redox signaling to the antioxidant cytoprotective responses mediated by Nfe2l1 and/or Nfe2l2 for maintaining a robust steady-state of redox homeostasis. Lastly, in view of such mutual interregulation of between Nfe2l1 and Nfe2l2 in different experimental cell lines, we should have to take severe cautions to interpret the relevant experimental results obtained from loss or gain of *Nfe2l1*, *Nfe2l2* alone, or both.

## 5. Materials and Methods

### 5.1. Chemicals, Antibodies, and Other Reagents

All chemicals were of the highest quality commercially available. The *tert*-butylhydroquinone (tBHQ) was from Sangon Biotech (Shanghai, China). Specific antibodies against Nfe2l1 were made in our own laboratory [42], whilst other antibodies against *α*-Pal^NRF1^ or V5 ectope were from Abcam and Invitrogen, respectively. Besides, both *β*-actin and secondary antibodies were from ZSGB-BIO (Beijing, China). Of note, the detailed information about other antibodies, as well as all other key reagents and resources used in this study, was all shown in Table S1.

### 5.2. Expression Constructs, Reporter Plasmids, and Other Oligos Used for sgRNA or siRNA

Besides four expression constructs for human and mouse Nfe2l1 or Nfe2l2 saved by our group [42, 53], another four expression constructs for human and mouse Pitx2, and *α*-pal^NRF1^, were here made by cloning each of those full-length cDNA sequences into the pcDNA3.1 vector. Particularly, the CRISPR/Cas9 plasmid constructs containing guide RNAs specifically targeting mouse *α*-pal^NRF1^ were created to generate a heterogeneous (*α-pal^NRF1+/-^*) knockout cell line from MEFs. Such sgRNAs (listed in Table S1) were designed with highly specific targets for precision genomic positions (as shown in Figure S1), before being employed in the sgRNA-directed gene-editing of *α-pal^NRF1^.*

Furthermore, several specific *cis*-regulatory luciferase reporter plasmids were prepared from cloning the indicated gene promoter regions. The mouse *Nfe2l1* promoter region (−2047 to +1210) was amplified by PCR from its genomic loci and then inserted into the pGL3-Basic vector. Another group of the *cis*-regulatory consensus, e.g., ARE and PitxRE- (*Pitx2* response elements-) adjoining sequences were cloned from the indicated gene promoter regions of, such as *Nfe2l1*, *TFAM*, and *α-pal^NRF1^* and then inserted into the pGL3-Promoter vector. In addition to those intact reporter genes, such as *PitxRE*-Luc and *ARE*-Luc, their relevant point-mutant reporters were also engineered herein. The fidelity of the above-described constructs was all confirmed to be true by sequencing. All these primers in the above gene manufacture, and other oligos for siRNA-mediated knockdown of the indicated genes, were listed in Table S1, which all were synthesized by Tsingke (Chengdu, China).

### 5.3. Cell Lines, Culture, and Transfection

Wild-type mouse embryonic fibroblasts (MEFs) were given as a gift from Akira Kobayashi. Their relevant knockout MEF lines, e.g., *Nfe2l1^−/−^*, *Nfe2l2^−/−^*, and *Keap1^−/−^*, were also obtained from the groups of Profs. Kobayashi and Hayes. Of note, all of these cell lines were originally prepared from Prof. Yamamoto's laboratory. But, another heterogeneous knockout line of *α-pal^NRF1+/-^* in the mouse was established by its gene-editing in our own laboratory as described above. Besides, the human wild-type (i.e., *hNfe2l1/2^+/+^*) hepatocellular carcinoma (HepG2) cells were originally from the American Type Culture Collection (ATCC, Manassas, VA, USA). The fidelity was conformed to be true by its authentication profiling and STR (short tandem repeat) typing map (by Shanghai Biowing Applied Biotechnology Co., Ltd) [55]. On this base, both *hNfe2l1α^−/−^* and *hNfe2l2^-/-ΔTA^* were previously established and characterized in our own laboratory [44]. These experimental cell lines were maintained for growth in Dulbecco's Modified Eagle's Medium (DMEM) supplemented with 5 mM glutamine, 10% (*v*/*v*) fetal bovine serum (FBS), and 100 units/mL penicillin-streptomycin, in the 37°C incubator with 5% CO_2_. Subsequently, the indicated cell lines were subjected to transfection for 8 h, which was performed by using Lipofectamine 3000 (Invitrogen, Carlsbad, CA, USA) containing different combinations of indicated plasmids, and then allowed for 24 h recovery from transfection in a fresh medium before relevant experimentation. In addition, two *Nfe2l1α*-inducible and *Nfe2l2*-inducible cell lines, along with their control cells, were described by Wang et al. [47].

### 5.4. Real-Time qPCR Analysis

Equal amounts of experimental cells were subjected to isolation of total RNAs by using the RNA simple Kit (Tiangen Biotech Co., Beijing, China). Then, 500 ng of total RNAs was added in a reverse-transcriptase reaction to generate the first strand of cDNA (with the Revert Aid First Strand Synthesis Kit from Thermo, Waltham, MA, USA). The synthesized cDNA fragments were served as the template for qPCR, in the GoTaq®qPCR Master Mix (from Promega), before being deactivated at 95°C for 10 min, and then amplified by 40 reaction cycles of the annealing at 95°C for 15 s and then extending at 60°C for 30 s. The final melting curve was validated to examine the amplification quality, whereas the mRNA expression level of *β*-actin served here as an optimal internal standard control. All the primers used for qPCR (Table S1**)** were synthesized by Tsingke (Chengdu, China).

### 5.5. Western Blotting Analysis

Experimental cells were harvested in a lysis buffer (0.5% SDS, 0.04 mol/L DTT, pH 7.5), which was supplemented with the protease inhibitor cOmplete Tablets EASYpack. The lysates were then denatured immediately at 100°C for 10 min, sonicated sufficiently, and diluted in 3 × loading buffer (187.5 mmol/L Tris-HCl, pH 6.8, 6% SDS, 30% Glycerol, 150 mmol/L DTT, and 0.3% Bromphenol Blue) at 100°C for 5 min. Thereafter, equal amounts of protein extracts were subjected to separation by SDS-PAGE containing 5–12% polyacrylamide and subsequent visualization by immunoblotting with distinct primary antibodies as indicated (Table S1). On some occasions, the blotted membranes were stripped for 30 min and then were reprobed with additional primary antibodies. Here, *β*-actin served as an internal control to verify equal loading of proteins in each of electrophoretic wells.

### 5.6. Distinct Cis-Regulatory Reporter Gene Assays

Equal numbers (1.4 × 10^5^) of indicated experimental cells were plated into 12-well plates. When allowed for growth to the density reaching 70–80% cell confluence, they were then transfected with Lipofectamine 3000 reagent (Invitrogen). For distinct lengths of the gene promoter studies, the pGL3-Basic vector containing 5 kb (−2248 to +2777), 2.7 kb (−2248 to +483), or 3 kb (−259 to +2777) of human *Nfe2l1* promoter, or 3.2 kb (−2047 to +1210) of mouse *Nfe2l1* promoter, or an empty pGL3-Basic vector as a blank control was cotransfected for 8 h with an indicated expression construct or an empty pcDNA3.1 as a control, plus another internal control pRL-TK reporter. Subsequently, the cells were allowed for a recovery from transfection in a fresh complete medium for 24 h, before they were lysed in a passive lysis buffer (E1910, Promega, Madison, WI, USA) for dual-luciferase assays. About 20 *μ*L of the supernatant of cells was assayed for the luciferase activity. Both firefly and renilla luciferase activities in each of the same samples were measured by the dual-luciferase reporter assay system (Promega). For the short consensus sequences of the indicated transcription factor-binding sites, the specific driven luciferase plasmids or corresponding paired point-mutants were cotransfected with the internal control pRL-TK, together with an expression construct for Nrf1, Nrf2, Pitx2, or an empty pcDNA3.1. All the resultant data were normalized and calculated as a fold change (mean ± S.D) relative to the activity of the control group (at a given value of 1.0).

### 5.7. Statistical Analysis

The statistical significance of changes was determined using the *Student'st*-test and/or *Multiple Analysis of Variations* (MANOVA). All the data presented in this study are shown as a fold change (mean ± S.D), each of which represents at least 3 independent experiments undertaken on separate occasions that were each performed in triplicate. In addition, statistical analysis of RNA-sequencing data was carried out as described by Wang et al. [47].

## Data Availability

The data that support the findings of this study are available from the corresponding author upon reasonable request.

## Supplementary Materials

Supplementary materials Figure S1: two specific constructs for gene-editing of mouse *α*-pal^NRF1^. Figure S2: two distinct models for integrative regulation of cellular respiratory and antioxidant gene transcription networks. Table S1: all the key reagents and resources used in this study. Table S2: distinct DNA-binding sites within 5-kbp of mouse gene promoter regions. Table S3: distinct DNA-binding sites within 5-kbp of human gene promoter regions.
