# Supplementary material for: Synergism and Antagonism of Two Distinct, but Confused, Nrf1 Factors in Integral Regulation of the Nuclear-to-Mitochondrial Respiratory and Antioxidant Transcription Networks
Source: Oxid Med Cell Longev. 2020 Nov 16;2020:5097109. doi: 10.1155/2020/5097109 (PMC7744186; doi:10.1155/2020/5097109)
Supplement: Supplementary materials — Figure S1: two specific constructs for gene-editing of mouse α-palNRF1. Figure S2: two distinct models for integrative regulation of cellular respiratory and antioxidant gene transcription networks. Table S1: all the key reagents and resources used in this study. Table S2: distinct DNA-binding sites within 5-kbp of mouse gene promoter regions. Table S3: distinct DNA-binding sites within 5-kbp of human gene promoter regions. [file 5097109.f1.docx]

**Supplementary Materials to support**

**"Synergism and antagonism of two distinct, but confused, Nrf1 factors in integral regulation of**

**the nuclear-to-mitochondrial respiratory and antioxidant transcription networks"**

**Shuwei Zhang^1,§^, Yangxu Deng^1,§^, Yuancai Xiang^1,2^, Shaofan Hu^1^, Lu Qiu^1,3^, and Yiguo Zhang^1^***

**^1^**The Laboratory of Cell Biochemistry and Topogenetic Regulation, College of Bioengineering and Faculty of Sciences, Chongqing University, No. 174 Shazheng Street, Shapingba District, Chongqing 400044, China.

**^2^**Department of Biochemistry and Molecular Biology, Collage of Basic Medical Sciences, Southwest Medical University, Sichuan, 646000, China.

**^3^**School of Life Sciences, Zhengzhou University, No. 100 Kexue Avenue, Zhengzhou 450001, Henan, China.

**^§^**Contributed equally to this work.

*Correspondence should be addressed to Yiguo Zhang (Email: [yiguozhang@cqu.edu.cn](mailto:yiguozhang@cqu.edu.cn), or [eaglezhang64@gmail.com](mailto:eaglezhang64@gmail.com))

**Figure S1.** **Two specific constructs for gene-editing of mouse *α-pal^NRF1^***


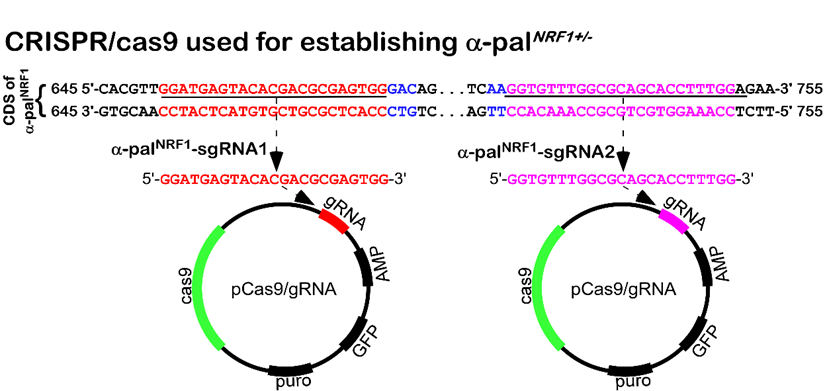


**Table S1. All the key reagents and resources used in this study.**

| **Reagent or Resource** | **Source** | | | **Identifier** | | |
| --- | --- | --- | --- | --- | --- | --- |
| **Oligonucleotides for sgRNA** | |  | | |  | |
| Mus_α-pal^NRF1^ sgRNA1-F  Mus_α-pal^NRF1^ sgRNA1-R  Mus_α-pal^NRF1^ sgRNA2-F  Mus_α-pal^NRF1^ sgRNA2-R | Tsingke  Tsingke  Tsingke  Tsingke | | ACTCACTATAGGGGATGAGTACACGACGCGAG  CTCTAAAACCTCGCGTCGTGTACTCATCC  ACTCACTATAGGGGTGTTTGGCGCAGCACCTT  CTCTAAAACAAGGTGCTGCGCCAAACACC | | |  |

| **Oligonucleotides for siRNA** |  | |  |
| --- | --- | --- | --- |
| si_homo_α-pal^NRF1^-F  si_homo_α-pal^NRF1^-R  si_homo_Pitx2-1-F  si_homo_Pitx2-1-R  si_homo_Pitx2-2-F  si_homo_Pitx2-2-R  si_homo_Pitx2-3-F  si_homo_Pitx2-3-R | Tsingke  Tsingke  Tsingke  Tsingke  Tsingke  Tsingke  Tsingke  Tsingke | AGCACUUCAUGGAGUCCAAdTdT  UUGGACUCCAGUAAGUGCUdTdT  GGAUGUAUAUAUAUCGAAAdTdT  UUUCGAUAUAUAUACAUCCdTdT  CGGACAUGUCCACACGCGAdTdT  UCGCGUGUGGACAUGUCCGdTdT  GGCCGAGCUAUGCAAGAAUdTdT  AUUCUUGCAUAGCUCGGCCdTdT | |

| **Oligonucleotides for construct** |  | | |  |
| --- | --- | --- | --- | --- |
| Mus_Pitx2-F  Mus_Pitx2-R  Mus_α-pal^NRF1^-F  Mus_α-pal^NRF1^-R  Homo_Pitx2-F  Homo_Pitx2-R  Homo_α-pal^NRF1^-F  Homo_α-pal^NRF1^-R  Mus_Nfe2l1-luc F  Mus_Nfe2l1-luc R  Mus_α-pal^NRF1^-ARE1w-luc F  Mus_α-pal^NRF1^-ARE1w-luc R  Mus_α-pal^NRF1^-ARE1m-luc F  Mus_α-pal^NRF1^-ARE1m-luc R  Mus_α-pal^NRF1^-ARE2w-luc F  Mus_α-pal^NRF1^-ARE2w-luc R  Mus_α-pal^NRF1^-ARE2m-luc F  Mus_α-pal^NRF1^-ARE2m-luc R  Mus_α-pal^NRF1^-ARE3w-luc F  Mus_α-pal^NRF1^-ARE3w-luc R  Mus_α-pal^NRF1^-ARE3m-luc F  Mus_α-pal^NRF1^-ARE3m-luc R  Mus_α-pal^NRF1^-ARE4w-luc F  Mus_α-pal^NRF1^-ARE4w-luc R  Mus_α-pal^NRF1^-ARE4m-luc F  Mus_α-pal^NRF1^-ARE4m-luc R  Mus_α-pal^NRF1^-ARE5w-luc F  Mus_α-pal^NRF1^-ARE5w-luc R  Mus_α-pal^NRF1^-ARE5m-luc F  Mus_α-pal^NRF1^-ARE5m-luc R  Mus_α-pal^NRF1^-ARE6w-luc F  Mus_α-pal^NRF1^-ARE6w-luc R  Mus_α-pal^NRF1^-ARE6m-luc F  Mus_α-pal^NRF1^-ARE6m-luc R  Homo_Nfe2l1-Pitx2RE1-luc F  Homo_Nfe2l1-Pitx2RE1-luc R  Homo_Nfe2l1-Pitx2RE1m-luc F  Homo_Nfe2l1-Pitx2RE1m-luc R  Homo_Nfe2l1-Pitx2RE2-luc F  Homo_Nfe2l1-Pitx2RE2-luc R  Homo_Nfe2l1-Pitx2RE2m-luc F  Homo_Nfe2l1-Pitx2RE2m-luc R  Homo_Nfe2l1-Pitx2RE3-luc F  Homo_Nfe2l1-Pitx2RE3-luc R  Homo_Nfe2l1-Pitx2RE3m-luc F  Homo_Nfe2l1-Pitx2RE3m-luc R  Homo_Nfe2l1-Pitx2RE4-luc F  Homo_Nfe2l1-Pitx2RE4-luc R  Homo_Nfe2l1-Pitx2RE4m-luc F  Homo_Nfe2l1-Pitx2RE4m-luc R  Homo_TFAM-ARE1-luc F  Homo_TFAM-ARE1-luc R  Homo_TFAM-ARE2-luc F  Homo_TFAM-ARE2-luc R  Homo_TFAM-ARE3-luc F  Homo_TFAM-ARE3-luc R  Homo_TFAM-ARE4-luc F  Homo_TFAM-ARE4-luc R  Homo_TFAM-Pitx2RE-luc F  Homo_TFAM-Pitx2RE-luc R | Tsingke  Tsingke  Tsingke  Tsingke  Tsingke  Tsingke  Tsingke  Tsingke  Tsingke  Tsingke  Tsingke  Tsingke  Tsingke  Tsingke  Tsingke  Tsingke  Tsingke  Tsingke  Tsingke  Tsingke  Tsingke  Tsingke  Tsingke  Tsingke  Tsingke  Tsingke  Tsingke  Tsingke  Tsingke  Tsingke  Tsingke  Tsingke  Tsingke  Tsingke  Tsingke  Tsingke  Tsingke  Tsingke  Tsingke  Tsingke  Tsingke  Tsingke  Tsingke  Tsingke  Tsingke  Tsingke  Tsingke  Tsingke  Tsingke  Tsingke  Tsingke  Tsingke  Tsingke  Tsingke  Tsingke  Tsingke  Tsingke  Tsingke  Tsingke  Tsingke | | GGGGTACCATGAACTGCATGAAAGGCCCGCTG  GCTCTAGA CA CACCGGCCGGTCGACTGCATA  GGGGTACCATGGAGGAGCACGGAGTGACCCAAACTG  GCTCTAGACACTGTTCCAAGGTCACCACCTCCACAGC  GGGGTACCATGAACTGCATGAAAGGCCCGCTTC  GCTCTAGACACACGGGCCGGTCCACTGCATA  GGGGTACCATGGAGGAACACGGAGTGACCCAAACCG  GCTCTAGACA CTGTTCCAATGTCACCACCTCCACAGC  CGACGCGTGCCAGGTAAGGACTGAGGAATG  CCCTCGAGTCGGGCAGAGAGTAGGAAACAC  CCGGGTGCTCTTACCTGCTGAGCCATCTCACCAGCCCCAGTGGTAGTCTTG  CTAGCAAGACTACCACTGGGGCTGGTGAGATGGCTCAGCAGGTAAGAGCACCCGGGTAC  CCGGGTGCTCTTACCTGCTGATTCATCTCACCAGCCCCAGTGGTAGTCTTG  CTAGCAAGACTACCACTGGGGCTGGTGAGATGAATCAGCAGGTAAGAGCACCCGGGTAC  CATCGTTGAGACATCTTTCCAGCCCCCTCAAACTAGAGTCTTAACGTTTAG  CTAGCTAAACGTTAAGACTCTAGTTTGAGGGGGCTGGAAAGATGTCTCAACGATGGTAC  CATCGTTGAGACATCTTTCCATTCCCCTCAAACTAGAGTCTTAACGTTTAG  CTAGCTAAACGTTAAGACTCTAGTTTGAGGGGAATGGAAAGATGTCTCAACGATGGTAC  CTGTAATGATGCATTGATACATGACCTGGCTGGACTGCCTCTTTGAATGTG  CTAGCACATTCAAAGAGGCAGTCCAGCCAGGTCATGTATCAATGCATCATTACAGGTAC  CTGTAATGATGCATTGATACATGACCTGAATGGACTGCCTCTTTGAATGTG  CTAGCACATTCAAAGAGGCAGTCCATTCAGGTCATGTATCAATGCATCATTACAGGTAC  CCAAACTTTATCATCTAATGAGCTCAGTCAGATCTAATCTCAGGTGGGTAG  CTAGCTACCCACCTGAGATTAGATCTGACTGAGCTCATTAGATGATAAAGTTTGGGTAC  CCAAACTTTATCATCTAATGATTTCAGTCAGATCTAATCTCAGGTGGGTAG  CTAGCTACCCACCTGAGATTAGATCTGACTGAAATCATTAGATGATAAAGTTTGGGTAC  CGCCCACCTTCTTGCAAGTTGTGAGGAGGCGCCTAGGCGGCAGTTCGAGGG  CTAGCCCTCGAACTGCCGCCTAGGCGCCTCCTCACAACTTGCAAGAAGGTGGGCGGTAC  CGCCCACCTTCTTGCAAGTTGTGAGGAGAAGCCTAGGCGGCAGTTCGAGGG  CTAGCCCTCGAACTGCCGCCTAGGCTTCTCCTCACAACTTGCAAGAAGGTGGGCGGTAC  CTTCTCATTTAACCAGAACCAGCCAACTCAAATTAGGTGTTTTCACAAAAG  CTAGCTTTTGTGAAAACACCTAATTTGAGTTGGCTGGTTCTGGTTAAATGAGAAGGTAC  CTTCTCATTTAACCAGAACCATTCAACTCAAATTAGGTGTTTTCACAAAAG  CTAGCTTTTGTGAAAACACCTAATTTGAGTTGAATGGTTCTGGTTAAATGAGAAGGTAC  CTTGTAGGGTGGGTGGAGAGAGGATTATTCAAGACAGGGCCTTATACG  CTAGCGTATAAGGCCCTGTCTTGAATAATCCTCTCTCCACCCACCCTACAAGGTAC  CTTGTAGGGTGGGTGGAGAGATTTTTTTTCAAGACAGGGCCTTATACG  CTAGCGTATAAGGCCCTGTCTTGAAAAAAAATCTCTCCACCCACCCTACAAGGTAC  CGGTGCAGTGGCTCACGCCTGTAATCCCAGCACTTTGGGAGGCTGAGG  CTAGCCTCAGCCTCCCAAAGTGCTGGGATTACAGGCGTGAGCCACTGCACCGGTAC  CGGTGCAGTGGCTCACGCCTGAAAAAACAGCACTTTGGGAGGCTGAGG  CTAGCCTCAGCCTCCCAAAGTGCTGTTTTTTCAGGCGTGAGCCACTGCACCGGTAC  CTCTATCTAATCTATTCCCCTGGATTAGATGAGGTTACAGAGTAAGAG  CTAGCTCTTACTCTGTAACCTCATCTAATCCAGGGGAATAGATTAGATAGAGGTAC  CTCTATCTAATCTATTCCCCTTTTTTTGATGAGGTTACAGAGTAAGAG  CTAGCTCTTACTCTGTAACCTCATCAAAAAAAGGGGAATAGATTAGATAGAGGTAC  CAGAGTAAGAAATACTTATATGGATTAGCAAAACTTAAAGACATGTTG  CTAGCAACATGTCTTTAAGTTTTGCTAATCCATATAAGTATTTCTTACTCTGGTAC  CAGAGTAAGAAATACTTATATTTTTTTGCAAAACTTAAAGACATGTTG  CTAGCAACATGTCTTTAAGTTTTGCAAAAAAATATAAGTATTTCTTACTCTGGTAC  CAACGCCGGGTTGGGGTGAGGCCGCCGCCGCGGTCCCTCCC  TCGAGGGAGGGACCGCGGCGGCGGCCTCACCCCAACCCGGCGTTGGTAC  CTCCAACAGAAGAAAGGCTGTCTCAGAAGGTGGTTAGCTCC  TCGAGGAGCTAACCACCTTCTGAGACAGCCTTTCTTCTGTTGGAGGTAC  CCTTGTCCTACCTCCTGCTGACTCATAGAATCAGCTTTAAC  TCGAGTTAAAGCTGATTCTATGAGTCAGCAGGAGGTAGGACAAGGGTAC  CCTCTTACTTTTGCACGCACACTCATGCATATGTGTATGTC  TCGAGACATACACATATGCATGAGTGTGCGTGCAAAAGTAAGAGGGTAC  CTTAGAGGATTTATGAAAGGATTAGTGATAATGTATGTAAAC  TCGAGTTTACATACATTATCACTAATCCTTTCATAAATCCTCTAAGGTAC | |
| **Oligonucleotides for qPCR** |  | | |  |
| Mus_Nfe2l1-F  Mus_Nfe2l1-R  Mus_Nfe2l2-F  Mus_Nfe2l2-R  Mus_HO-1-F  Mus_HO-1-R  Mus_GCLM-F  Mus_GCLM-R  Mus_Aldh1a1-F  Mus_Aldh1a1-R  Mus_COX5A-F  Mus_COX5A-R  Mus_Gsta1-F  Mus_Gsta1-R  Mus_Gstπ-F  Mus_Gstπ-R  Mus_MT1-F  Mus_MT1-R  Mus_α-palNRF1-F  Mus_α-palNRF1-R  Mus_SOD1-F  Mus_SOD1-R  Mus_TFAM-F  Mus_TFAM-R  Mus_β-actin-F  Mus_β-actin-R  Homo_Nfe2l1-F  Homo_Nfe2l1-R  Homo_Nfe2l2-F  Homo_Nfe2l2-R  Homo_HO-1-F  Homo_HO-1-R  Homo_GCLM-F  Homo_GCLM-R  Homo_Aldh1a1-F  Homo_Aldh1a1-R  Homo_COX5A-F  Homo_COX5A-R  Homo_Gstπ-F  Homo_Gstπ-R  Homo_MT1E-F  Homo_MT1E-R  Homo_α-palNRF1-F  Homo_α-palNRF1-R  Homo_SOD1-F  Homo_SOD1-R  Homo_TFAM-F  Homo_TFAM-R  Homo_Pitx2-F  Homo_Pitx2-R  Homo_β-actin-F  Homo_β-actin-R | Tsingke  Tsingke  Tsingke  Tsingke  Tsingke  Tsingke  Tsingke  Tsingke  Tsingke  Tsingke  Tsingke  Tsingke  Tsingke  Tsingke  Tsingke  Tsingke  Tsingke  Tsingke  Tsingke  Tsingke  Tsingke  Tsingke  Tsingke  Tsingke  Tsingke  Tsingke  Tsingke  Tsingke  Tsingke  Tsingke  Tsingke  Tsingke  Tsingke  Tsingke  Tsingke  Tsingke  Tsingke  Tsingke  Tsingke  Tsingke  Tsingke  Tsingke  Tsingke  Tsingke  Tsingke  Tsingke  Tsingke  Tsingke  Tsingke  Tsingke  Tsingke  Tsingke | TGAATGTGGCTTTCGCTCC  GTGAAGTAATTGTCCAGGTCTATGC  GAGAGGTAAGAATAAAGTCGC  GTAGATGGAGGTTTCTGTCGT  GGAATTTATGCCATGTAAATGC  CCTGCAGCTCCTCAAACAGCTC  AGGAGCTTCGGGACTGTATCC  GGGACATGGTGCATTCCAAAA  CCAGTTCTTAACCCTGCAACTGAG  GCCAATCTGGAAAGCCTGTCTTG  TGTCTGTTCCATTCGCTGCTATTC  TTACGCAATTCCCAGGCATCAATG  CAGGGGTGGAGTTTGAAGAGAAG  AGTTGAGAATGGCTCTGGTCTGC  GGAACTTCTTCTCTATGT  ACTAAAACTTGAAAACCT  ACCTCCTGCAAGAAGAGCTG  GCTGGGTTGGTCCGATACTA  CCCCCGAGGACACTTCTTATGATG  GGCCGTTTCCGTTTCTTCCCTGTT  CGGATGAAGAGAGGCATGTT  CACCTTTGCCCAAGTCATCT  CGCATCCCCTCGTCTATCAGTC  TAAATTTGGGTAGCTGTTCTGTGG  GGCTGTATTCCCCTCCATCG  CCAGTTGGTAACAATGCCATGT  GCTGGACACCATCCTGAATC  CCTTCTGCTTCATCTGTCGC  TCAGCGACGGAAAGAGTATGA  CCACTGGTTTCTGACTGGATGT  CAGAGCCTGGAAGACACCCTAA  AAACCACCCCAACCCTGCTAT  GTGTGATGCCACCAGATTTGAC  CACAATGACCGAATACCGCAGT  TGAATGGCATGATTCAGTGAGTGG  CCAATCTGAAAAGCCTGTCTTGCG  TCCAGTCAGTTCGCTGCTATTCC  CTTTACGCAATTCCCAGGCATC  TGATGGGGAGGTTCACGTAC  GGGAGGCAAGACCTTCATTG  ATGGACCCCAACTGCTCTTGCGCCA  ACAGCAGCTGCACTTCTCCGATG  CTTCTCCCGAGGACACCTCTTAC  CGTTTCTTTCCTGTTGCCACAGC  AGTGCAGGGCATCATCAATTTCG  GGTACAGCCTGCTGTATTATCTCC  CCCCTTCAGTTTTGTGTATTTACCG  TAGTTTTTGCATCTGGGTTCTGAGC  ACTTTACCAGCCAGCAGCTC  GGGTACATGTCGTCGTAGGG  CATGTACGTTGCTATCCAGGC  CTCCTTAATGTCACGCACGAT | | |

| **Antibodies** |  |  | | |
| --- | --- | --- | --- | --- |
| Nfe2l1  Nfe2l2  Keap1  HO-1  GCLM  α-palNRF1  TFAM  SOD1  Aldh1a1  b-actin  Alexa Fluor 488 - Conjugated Goat anti-rabbit IgG | Zhang’s [1]  Abcam  Sangon Biotech  Abcam  Abcam  Abcam  Proteintech  Proteintech  Abcam  ZSGB-BIO  ZSGB-BIO | N/A  ab62352  D154142  ab52947  ab126704  ab175932  19998-1-AP  10269-1-AP  ab52492  TA-09  ZF-0511 | | |
| **Chemicals** |  |  | | |
| tert-Butylhydroquinone (tBHQ) | Sigma Aldrich | 112941 | | |
| **Recombinant DNA** |  | | |  |
| Homo_Nfe2l1-luc  Homo_Nfe2l1-luc1  Homo_Nfe2l1-luc2  pARE-luc  pcDNA3.1  pGL3-Basic  pGL3-promoter  pRL-TK | Qiu’s[2]  Qiu’s[2]  Qiu’s[2]  Zhang’s [1]  invitrogen  Promega  Promega  Promega | | N/A  N/A  N/A  N/A  V79020  VQP0121  VQP0124  VQP0126 | |
| Software and Algorithms |  | |  | |
| Canvas X  Excel  Primer Premier 5 | Canvas GFX, Inc.  Microsoft  PREMIER Biosoft | | | <https://www.canvasgfx.com/>  <https://www.microsoft.com/>  [https://www.PremierBiosoft.com/](https://www.premierbiosoft.com/) |
| Others |  | | |  |
| Cas9/gRNA Construct Kit  Dual-luciferase reporter assay system  GoTaq® qPCR Master Mix  Lipofectamine® 3000 Transfection Kit  Revert Aid First Strand Synthesis Kit  RNAsimple Total RNA Kit | v-solid  Promega  Promega  Invitrogen  Thermo  Tiangen Biotech | | | VK001  E1910  A6001  L3000-015  K1622  DP419 |

Reference:

[1] Zhang, Y.G.; Hayes, J.D. Identification of topological determinants in the N-terminal domain of transcription factor Nrf1 that control its orientation in the endoplasmic reticulum membrane. Biochem. J. 2010, 430, 497–510.

[2] Qiu, L.; Wang, M.; Hu, S.; Ru, X.; Ren, Y.; Zhang, Z.; Yu, S.; Zhang, Y. Oncogenic activation of Nrf2, though as a master antioxidant transcription factor, liberated by specific knockout of the full-length Nrf1alpha that acts as a dominant tumor repressor. Cancers 2018, 10, 520.

**Figure S2.**

**Two distinct models for integrative regulation of cellular respiratory and antioxidant gene transcription networks.**

(A) A model is proposed on the base of experimental evidence obtained from MEFs, to give a better understanding of regulatory cross-talks among Nfe2l1^Nrf1^, Nfe2l2^Nrf2^ and α-Pal^NRF1^, along with Pitx2, which are responsible for the nuclear-to-mitochondrial respiratory and antioxidant gene transcription networks. Of note, Nfe2l1^Nrf1^ can make opposing contributions to the bi-directional regulation of Nfe2l2^Nrf2^ and α-Pal^NRF1^ by itself and target proteasome (*PSM*) at two distinct layers. Nfe2l2^Nrf2^ can also determine putative post-transcriptional regulation of Nfe2l1^Nrf1^ and α-Pal^NRF1^, but the detailed mechanism remains unclear. In turn, α-Pal^NRF1^ has a capability to positively regulate Nfe2l1^Nrf1^ and Nfe2l2^Nrf2^, albeit none of canonic GC-rich α-Pal-binding sites exist within these mouse CNC-bZIP gene promoter regions (Table S2). Such the nucleus-controlled mitochondrial respiratory and oxidative phosphorylation is also a primary source of byproduct reactive oxygen species (ROS) in cells, which can trigger putative physiological activation of Nfe2l1^Nrf1^, Nfe2l2^Nrf2^ and α-Pal^NRF1^. Besides, GABP^NRF2^ is also required for this process, but not yet identified herein. In addition to negative regulation of Nfe2l2^Nrf2^, Keap1 can contribute to rather positive regulation of this CNC-bZIP transcription. (B) Another model is proposed, based on experimental evidence obtained from HepG2 cells, to give a better explanation of distinct cross-talks amongst Nfe2l1^Nrf1^, Nfe2l2^Nrf2^ and α-Pal^NRF1^, along with Pitx2. All these factors are converged on the nucleus-encoded mitochondria-targeting TFAM, but Nfe2l2^Nrf2^ may contribute to dual opposing effects on this mitochondrial transcription factor. Furthermore, α-Pal^NRF1^ can make a negative contribution to transcription of human Nfe2l1^Nrf1^ and Nfe2l2^Nrf2^, albeit all three factors are activated by redox inducer tBHQ. Of note, Nfe2l2^Nrf2^ is dominantly negatively regulated by Nfe2l1^Nrf1^. In addition, human Nfe2l1^Nrf1^ is also essential for stabilization of Keap1, but whether the adaptor subunit of Cullin 3-based E3 ubiquitin ligase is involved in the proteolytic processing of Nfe2l1^Nrf1^ remains elusive.


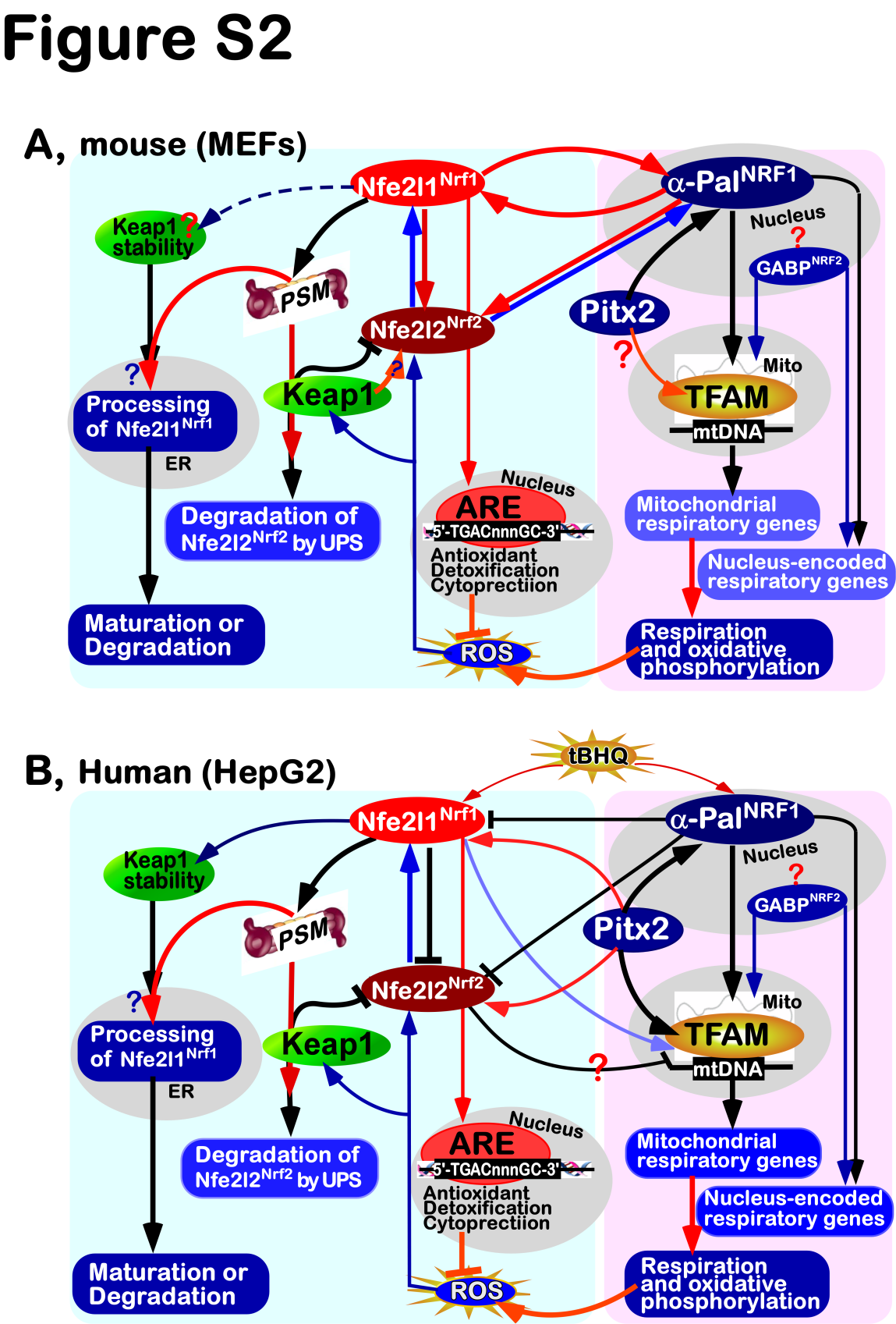


| **Gene ID** | **Name** | **ARE/EpRE (TGAC/GnnnGC）** | **α-PAl-NRE (GCGCnnGCGC)** | **PitxRE (TAATCC)** | **GABPα-binding site (GGAAnnGGAA)** |
| --- | --- | --- | --- | --- | --- |
| **18023** | **Nfe2l1^Nrf1^** | ACTGAGCCATCTCACCAGC (-3590 to -3572)  GGCCCTGACTGCGCTGTGA (-2915 to -2897)  ACTGAGCCATCTCACCAGC (-2108 to -2090)  AACGAGCCAACTCAAGCCA (-1822 to -1804)  GAGGGTGAGTATGCCAGGT (-1593 to -1575)  GTACATGAGAATGCCCTTG (-1467 to -1449)  GGCCTGCACACTCATTGGA (-184 to -166) | NONE | CACCAGGATTAGCTGC (-4177 to -4162)  TGCCAGGATTAAAGGC (-1427 to -1412) | CTAGGTTCCATGCTTCCGTGTA (-1959 to -1938) |
| **18024** | **Nfe2l2^Nrf2^** | TGTGCTGAGTTGGCTGGGA (-4236 to -4218)  ATTGCTGAGATTGCTGAGG (-3632 to -3614)  ATACAGCCTGCTCAAATGT (-3541 to -3523)  ACCTGTGAGCTTGCTGGAA (-3015 to -2997)  TTGCCGCCGCCTCACCTCT (185 to 203) | NONE | ATAGGTAATCCTGTCA (-3174 to -3159) | AACCTTTCCTTTCCACTAT (-4695 to -4677)  CGAGGGGAACGCGGGAACTCCG (-191 to -170) |
| **18181** | **α-Pal^NRF-1^** | GCTGAGCCATCTCACCAGC (-4586 to -4568)  TTCCAGCCCCCTCAAACTA (-4345 to -4327)  ATACATGACCTGGCTGGAC (-4172 to -4154)  AATGAGCTCAGTCAGATCT (-3308 to -3290) | NONE | TGGCAGGATTAATATC (-4278 to -4263)  TGCTGGGATTAAAGGC (-1763 to -1748)  GTCTTTAATCCCAGAA (-1522 to -1507)  GCCTTTAATCCCAGCA (-1027 to -1012)  CCTTCGGATTAGCAAC (-270 to -255) | TCCCAGGAAAATGGAACTTAC (-4078 to -4058)  AGAAAGGAAGGAAGTAAGGAAGGAAGGAAGAGAA  (-1115 to -1082) |
| **8065** | **GABPα^NRF-2^** | GGGCTGCAGGCTCACCACC (-594 to -576) | NONE | TACTGGGATTAAAGGC (-4934 to -4919)  GTGTGTAATCCTCCAA (-4804 to -4789) | ATTTATTCCTTCCAACCC (-4465 to -4448)  TCATAGGAAGGAAAACAG (-3170 to -3153) |
| **17748** | **Mt1** | ACCTCTGACATAGCCTGTT (-3843 to -3825)  TTTGTTGACCAGGCTGGCC (-1595 to -1577)  GATGAGCCCCCTCAGGAGT (-366 to -348)  GCGCGTGACTATGCGTGGG (48 to 66) | NONE | ACCTTTAATCCCAGCA (-4774 to -4759) | TAGTTTTCCCTCTTCCACAGA (-4333 to -4313) |
| **20655** | **SOD1** | GCTGGTGAGATGGCTCAGT (-4753 to -4735)  GACAGGCATTCTCAGCCTG (-4008 to -3990)  ATTGAGCCATCTCACCAGC (-3654 to -3636)  GCTGTGCAAAGTCAGTGAC (-342 to -324)  GGTGGGCGTGGTCAGACTC (-61 to -43) | CCGGGGCGCGGGGCGCAGGCC (176 to 196) | TAAAATAATCCTTTTA (-3023 to -3008)  ACCTTTAATCCCAGCA (-2117 to -2102) | AAGAATTCCCTTCCTCTTA (-1809 to -1791)  TGGTCTTCCTTCCTCTTG (-647 to -630) |
| **14857** | **Gsta1** | GGTCGTGAGGAAGCACACA (-4179 to -4161)  ATCAATGACATTGCATGCC (-4130 to 4112)  GGAAATGACATTGCTAATG (-726 to -708)  AATGGTGACAAAGCAACTT (-711 to -693) | NONE | CCTCCTAATCCTTCCC (-2949 to -2934)  ACCAAGGATTAACTAA (-852 to -837) | CCATGGGAACTTGGAACCAAG (-1605 to -1585)  AGTGCTTCCTTTCCAGGGA (-1341 to -1323)  CGGGTTTCCTTCCCTCCT (-1186 to -1169) |
| **11668** | **ALdh1a1** | TGAGGGCAAAGTCATTTTT (-4408 to -4390)  AATAGTGAGTGAGCAGAAA (-4125 to -4107)  GTTCAGCATTGTCAGGTGG (-3689 to -3671)  AACTGTGAGTCTGCAAAAC (-1741 to -1723)  GAGAAGCTCAGTCAAGGCA (-1067 to -1049)  TGCCCTGAGTCTGCCCATC (-90 to -72) | NONE | ATCAGGGATTAAATAA (-5006 to -4991)  GTCTTTAATCCACAGT (-4303 to -4288) | GTAAGGGAAAGAGGAAAGAAA (-2714 to -2694)  TCTTGTTCCTTCCATATC (-922 to -905)  GTCCTTTCCAAGTTCCCAGAT (-840 to -820)  AAGCAGGAAAAGGGAATGGAAAAAAA (-320 to -295) |
| **21780** | **Tfam** | TTCCTGCGGACTCATGAAA (-381 to -363) | TTTCGGCGCCTAGGGCGCTCTCC (332 to 354) | GGCTGGGATTACAGGC (-684 to -669) | GAGGGGGAAGGGGAAGACCC (-4667 to -4648)  ATGAGGGAAGTAGGAAACATC (-2398 to -2378) |
| **224481** | **Tfb1m** | GTTGTGCCTGCTCACAGAC (-4029 to -4011)  CATCTTGACAATGCCTTTA (-3806 to -3788)  CTGCAGCCTGCTCAGAATT (-3506 to -3488)  ATTTTTGACTCAGCTCTAG (-2632 to -2614)  CCTGATGAGTAAGCCTTCC (-1230 to -1212)  GATGCTGACCTTGCTGGCA (-935 to -917) | NONE | TGAGTTAATCCAACAT (-4119 to -4104) | AAGCCTTCCGTTTCCACTCT (-1220 to -1201) |
| **15278** | **Tfb2m** | GCCTCTGACATAGCTAGGA (-4642 to -4624)  CCAATGCTGGGTCAGGCTG (-3842 to -3824)  GGAAATGACTGAGCCCTCT (-3324 to -3306)  TGGACTGAGTAGGCTGTGG (-1903 to -1885)  CGCGCTGACATGGCGGCCG (-281 to -263)  GGATCGCGCTGTCAGCGTT (177 to 195) | GGGGAGCGCGCGCGGAGA (-551 to -534)  AATTGGCGCGACGCGCGACAA (-533 to -513) | GGCTTTAATCCTTTTA (-845 to -830) | ACATGGGAAGGAATTTTA (-828 to -811)  TAGGCGGAAGCGGAAGCGAG (-35 to -16) |
| **12858** | **COX5α** | AAGCAGCCTTGTCATGGAA (-4022 to -4004)  AAGTCTGACAGGGCTACAG (-2627 to -2609)  AGAGATGACTTAGCTGCTC (-2459 to -2441)  GCTGGTGAGCCAGCTCCTT (-1382 to -1364)  TCTGATGACCAAGCATTCA (-1007 to -989)  TCCTCTGAGAGCGCAGAAA (-640 to -622) | CTTTGGCGCGGCGCCGACG (-128 to -110) | TGCTGGGATTAAAGGC (-4579 to -4564)  TTTTTTAATCCCTTTT (-4170 to -4155)  TGCTAGGATTAAAGGT (-2152 to -2137)  TCCTTTAATCCCAGCA (-1368 to -1353)  CCTTCTAATCCCTCTC (-1037 to -1022) | NONE |
| **17995** | **Ndufv1** | TTGACTGACTGAGCTGGCT (-3071 to -3053)  AGCTGGCTCGGTCAGATAA (-3060 to -3042)  CTGTCTGACAAAGCAGAAG (-865 to -847) | NONE | CAAAGGGATTAAATCA (-4799 to -4784)  ACTCCTAATCCTCCTG (-4635 to -4620)  TGCTGGGATTAAAGGC (-3280 to -3265) | GAAGAGGAATAGGAAGAAAA (-4328 to -4309)  CTATGGGAAGGAAACTAT (-2043 to -2026)  CAGAAGGAAGGAAGGAAAGAAA (-852 to -831)  CTGTAGGAAGTGGAATACTT (-208 to -189)  TACTAGGAAAAGGAAGAGTC (-154 to -135) |
| **230075** | **Ndufb6** | GTTGGTGAGAAAGCAGAGA (-3671 to -3653)  TAAAAGCATGGTCAGGCCT (-3384 to -3366)  GTTGGTGAGAAAGCAGAGA (-1659 to -1641)  TCTTCTGACGGAGCAGTAA (-1072 to -1054)  AAGAGTGAGCGAGCCTATG (-437 to -419) | NONE | GCCCTGGATTAGCTAT (-2245 to -2230)  GAAAATAATCCCTTAG (-1597 to -1582)  TGCTGGGATTAAAGGC (-4728 to -4713)  CCCACTAATCCTCTAT (-585 to -570) | AGTAGTTCCTTCCATTCT (-4133 to -4116)  AAGTCTTCCATATTCCTACTA (-3482 to -3462) |

**Table S2. Distinct DNA-binding sites within 5-kbp of mouse gene promoter regions**

| **Table S3. Distinct DNA-binding sites within 5-kbp of human gene promoter regions** | | | | | |
| --- | --- | --- | --- | --- | --- |
| **Gene ID** | **Name** | **ARE/EpRE (TGAC/GnnnGC）** | **a-PAl-NRE (GCGCnnGCGC)** | **PitxRE (TAATCC)** | **GABPa-Binding site (GGAAnnGGAA)** |
| **4779** | **Nfe2l1^Nrf1^** | TTACTGCACCCTCAACCTC(-3276 to -3258) TTTGTTGAGGAAGCATCTC(-1982 to -1964) TCACTGCAGCCTCAATCTC(-1934 to -1916) | AGAAAGCGCTGGAAGCGCCCAAC  (-308 to -286) | AGAGAGGATTATTCAA(-4657 to -5642)  GCCTGTAATCCCAGCA(-4230 to -4215)  CCCCTGGATTAGATGA(-3419 to -3404)  TATATGGATTAGCAAA(-3382 to -3367) | CAACGTTCCGCCAACGTTCCGCCAA(-668 to -644) ACACCTTCCTATTCCCTGAC(-351 to -332) GCACATTCCTTTCCCAGAA(170 to 188) |
| **4780** | **Nfe2l2^Nrf2^** | AGACAGCTTCCTCATTCCC(-3274 to -3256) GGTGTTGACTTAGCTTAGC(-3028 to -3010) | GAAGGGCGCCATCTGTGGCGTGGTG  GCTGCGCTTTGG(-393 to -357) | GCCTGTAATCCCAGCA(-4524 to -4509)  CCCTGGGATTATAGGT(-3593 to -3578)  TGCTGGGATTATAGGC(-2048 to -2033) | ACAGCTTCCTCATTCCCAGGG(-3272 to -3252) TCATCTTCCATGACTTTTCCCATGG(-2832 to -2808) |
| **4899** | **a-Pal^NRF-1^** | GTGGCGCTCCCTCAGGAGA(-5010 to -4992) CAGTGGCTCAGTCATGGCT(-4523 to -4505) GTGGTGCGATCTCAGCTCA(-4340 to -4322) TGGTAGCTAACTCATTCAA(-3868 to -3850) TCACTGCAGCCTCAGTCTC(-2794 to -2776) TATAGGCTGACTCAGTTTC(-2382 to -2364) GTGGCGCAGTCTCAGCTCA(-743 to -725) CTTTTTGAGACAGCCATTT(-493 to -475) | NONE | TGCTGGGATTACAAGC(-4132 to -4117)  TGCTGGGATTACAGGC(-3994 to -3979)  TGTTGGGATTACAGGC(-2607 to -2592)  ACCTTGGATTATTATT(-2100 to -2085)  TGCTGGGATTACAGGT(-539 to -524) | TGGGTTTCCCAGTTTCCCTTTC(-3024 to -3003) CCAAATTCCATAGCTTTCCATTAC(-2478 to -2455) GTTTAGGAAGAGCAGGAATGCCC(-2157 to -2135) |
| **2551** | **GABPa^NRF-2^** | TAAACTGACAGGGCTTGCC(-3696 to -3678) | CCCGTGCGCCCGGGGCGGGTAGGC  CTGGCCGAAAATCTCTCCCGCGCG  CCTGAC(-510 to -457) | GCCTGTAATCCCAGCA(-4556 to -4541)  GCCTGTAATCCCAGCT(-4411 to -4396)  AAAAATAATCCAAATT(-4119 to -4104)  GTGTGTAATCCCAGCA(-4035 to -4020)  GCCTGTAATCCCAGTT(-3903 to -3888)  TGTTGTAATCCCAGCA(-2295 to -2280)  GCCTGTAATCCCAGCT(-2162 to -2147)  ACTGAGGATTAGAAGT(-1799 to -1784)  GCTTGTAATCCCAGCA(-1750 to -1735)  ACACATAATCCCATAT(-979 to -964)  TCCTTTAATCCCTTTC(-947 to -932) | CCTCGTTCCGGGGCCTTTTCCCCCAC(611 to 636) |
| **4543** | **Mt1** | TGTTGTGACTTGGCTCTGA(-4954 to -4836) CACCCTGACAATGCAATAG(-3716 to -3698) GCGGTGCGGACTCAGCGGG(-98 to -80) | CCACCGCGCAGAGCTCAGGGGGTGG  TGCGCCCGGCCCTTCTGCGGCGCAC  AGC(-168 to -116) TGCAGGCGCGGAGCTGGGCCTCTG  CGCCCGGC(-74 to -43) | AATGTTAATCCCCAAG(-3625 to -3610) | ATGTGGGAAAGTTTGGAACTTCC(-4058 to -4036) ATTTCGGAAGTTGTATGGAAATGCA(-3313 to -3289) CTCGGGGAAACTGGGAAAGGCG(-274 to -253) |
| **6647** | **SOD1** | TCAGCGCCCTGTCAAAACA(-4794 to -4776) CTGCCTGAGCCAGCAGTGG(-4713 to -4695) ACTCCTGAGCCAGCGAGAC(-4461 to -4443) TCACTGCAGCCTCAACCTC(-2714 to -2696) TGAGCTGAGATTGCACCAC(-2024 to -2006) CTGGGTGACAGAGCGAGAC(-792 to -774) | NONE | ACCTGTAATCCCAGCA(-2229 to -2214)  GTCTGTAATCCCAGCA(-1031 to -1016) | TCTAATTCCTTCCCTCCTTCCCTCCT(-3021 to -2996) GGCCTTTCCTCCTTTCCCCACC(-2439 to -2418) |
| **2938** | **Gsta1** | TCTGTGCTATCTCACAGGC(-2087 to -2069) CAGCCTGAGGGAGCTTCCC(-1712 to -1694) | NONE | CTATGGGATTAGTCAG(-4550 to -4535)  TGTTTGGATTAGAAGT(-886 to -871) | TTCACTTCCAAGAAGCTTTCCTTGAT(-3932 to -3907) ATGTGTTCCTGATCTGTTTCCTCTGA(-2820 to -2795) GCCCTTTCCATCTGGTTCCTTCCTTATG(-2292 to -2265) AAGCCTTCCCTCAGTTCCTTCAG(-1011 to -989) GTGATTTCCAGGAATCCTTCCCTGCG(-872 to -847) |
| **216** | **ALdh1a1** | CTAGAGCTTTGTCACTTTC(-8168 to -8150) | NONE | TCCCATAATCCCCACA(-2878 to -2863)  AACCAGGATTACTTTC(-480 to -465) | ATTAAGGAAGCAAACTGGAAACTAG(-3855 to -3831) CTGCCTTCCACTTCCATTTT(-3470 to -3451) TTTGCTTCCCCTTCCACTGT(-2693 to -2674) GTGTGTTCCGAATTCCCTAAA(-401 to -381) |
| **7019** | **Tfam** | TGCACGCACACTCATGCAT(-4517 to -4499) CTCCTGCTGACTCATAGAA(-3758 to -3740) GAAAGGCTGTCTCAGAAGG(-763 to -745) TGGGGTGAGGCCGCCGCCG(-1 to 18) | CTCCCGCGCCTGCGCCAATT  (200 to 219) | TGAAAGGATTAGTGAT(-4044 to -4029) | TCTCTTTCCCTTCCTTCTT (-3540 to -3522) CATCTGGAAATTGGGAATAATG(-2268 to -2247) |
| **51106** | **Tfb1m** | TTTCTGCAATCTCATGTTA(-4396 to -4378) GACAATGAGCAGGCTTTCA(-3617 to -3599) TCTGGGCATTGTCAAATGA(-1301 to -1283) ATAATGCTTTGTCAGGTAT(-1183 to -1165) CGCATGCGCTCTCAGCACG(-132 to -114) | ACTTAGCGCATGCGCTCTCA  (-138 to -119) | ACTGAGGATTAGATAT(-2703 to -2688)  AAATGGGATTAGCAAC(-2403 to -2388)  TTAGCTAATCCCTTTT(-2366 to -2351)  AGCTGTAATCCTAGCA(-719 to -704) | AAGTGTTCCATGGTTCCCTCAG(-3551 to -3530) GAGCCGGAAGCCCTTGGGAACTTCA(-357 to -333) |
| **64216** | **Tfb2m** | AGGATGCAAAGTCACCTCT(-1554 to -1536) CCGCTTGACCTGGCCCGGA(14 to 32) | AATTGGCGCGACGCGCAGCAA  (-632 to -612) AGGAAGCGCAGAGCGCGGACA  (-166 to -146) | GCCTGTAATCCCAGCA(-2585 to -2570)  GCCTGTAATCCCAGCT(-2458 to -2443)  TGCTGGGATTACAGGC(-1791 to -1776) | CATCTTTCCTTTTAAATTCCCCTAA(-3916 to -3892) AGAGAGGAATGGAAGGGAG(-850 to -832) GAGGCTTCCGAAAGGCTTTCCATCCC(-473 to -448) GAGGCGGAAGCGGAAGTGAG(-61 to -42) |
| **9377** | **COX5a** | GTCTAGCTCTGTCACCCAG(-1985 to -1967) ATGGTGCGATCTCAACTCA(-873 to -855) CACAATGAGAAGGCGGGGC(-594 to -576) | CCACAGCGCCCGGGCGCAAAGT  (-265 to -244) | GCCTGTAATCCCAACA(-3617 to -3602)  TGCTGGGATTATAGGC(-2361 to -2346)  TGCTGGGATTACAGGC(-2069 to -2054)  TGCTAGGATTACAGGC(-1798 to -1783)  AGCTGGGATTACAGGC(-800 to -785)  TGCTAGGATTACAGGC(-665 to -650)  TGCTGGGATTACAGGC(-286 to -271) | AGGTAGGAAATGGCAGGAAGAGAT(-4829 to -4806) TCCTTTTCCCCGCCAGGTTCCTACCC(-1462 to -1437) |
| **4723** | **Ndufv1** | GCGCATGAGTGAGCCGAGA(-4654 to -4636) GACCAGCCTGGTCAACATG(-4523 to -4505) TACACTGAGATAGCAGGGT(-3993 to -3975) TTTGGTGAGCCAGCTCCTC(-3781 to -3763) AGCCATGAGGAAGCAGGTC(-3602 to -3584) AATACTGAGTATGCTGCAG(-3560 to -3542) TGAGTTGACACAGCACATG(-2703 to -2685) AGAGATGACAGGGCCACAG(-2142 to -2124) | CCGGTGCGCGGCGCTGGCG  (24 to 42) | GCCTGTAATCCCAACA(-4785 to -4770)  ACCTGTAATCCCAGCT(-4450 to -4435)  GTAAAGGATTAAGTGC(-3210 to -3195)  TTTACTAATCCTCCTC(-2979 to -2964)  GCCCTTAATCCATTCA(-2723 to -2708)  TATAATAATCCCACTG(-2239 to -2224)  TTGGAGGATTAAGGAG(-1709 to -1694)  AGCCCGGATTAGCTGT(-1442 to -1427) AGCCCGGATTAGCTGT(-1430 to -1415) | AGGCATTCCTTCCTCTTT(-2995 to -2978) GGTCTTTCCCTTCCCACGA(-2923 to -2905) TGCAGGGAATGTTTGGAAGGTAA(-2110 to -2088) GATGAGGAAGGAATCCAC(-1529 to -1512) ACTGTGGAAAGGAAGAAAG(-1203 to -1185) |
| **4712** | **Ndufb6** | GTTTAGCACCCTCATATAA(-4143 to -4125) ACAGATGAGACAGCTGCCC(-422 to -404) AGAGCTGAGAAGGCGATGG(172 to 190) | TAACCGCGCGCGGCGCTCGGC  (5 to 25) | TTTCCTAATCCGACCC(-4798 to -4783)  TGCTGGGATTACAGGC(-2343 to -2328) | AAAACTTCCCTGCTTTCCGACAT(-4848 to -4826) |
